# Global gene expression analysis of apple fruit development from the floral bud to ripe fruit

**DOI:** 10.1186/1471-2229-8-16

**Published:** 2008-02-17

**Authors:** Bart J Janssen, Kate Thodey, Robert J Schaffer, Rob Alba, Lena Balakrishnan, Rebecca Bishop, Judith H Bowen, Ross N Crowhurst, Andrew P Gleave, Susan Ledger, Steve McArtney, Franz B Pichler, Kimberley C Snowden, Shayna Ward

**Affiliations:** 1 The Horticulture and Food Research Institute of New Zealand Ltd., Mt Albert, Private Bag 92169, Auckland Mail Centre, Auckland 1142, New Zealand; 2 John Innes Centre, Colney Lane, Norwich NR4 7UH, UK; 3 Boyce Thompson Institute for Plant Research, Tower Road, Cornell University Campus, Ithaca, NY 14853, USA; 4 22 Ramphal Terrace, Khandallah, Wellington, New Zealand; 5 4 La Trobe Track, RD2 New Lynn, Karekare, Auckland, New Zealand; 6 Department of Horticultural Science, North Carolina State University, Mountain Horticultural Crops Research and Extension Centre, 455 Research Drive, Fletcher, NC 28732-9244, USA; 7 Microbial Ecology & Genomics Lab, School of Biological Sciences, University of Auckland, Auckland, New Zealand; 8 Monsanto Company – O3D, Product Safety Center, 800 North Lindbergh Blvd., St. Louis, MO 63167, USA

## Abstract

**Background:**

Apple fruit develop over a period of 150 days from anthesis to fully ripe. An array representing approximately 13000 genes (15726 oligonucleotides of 45–55 bases) designed from apple ESTs has been used to study gene expression over eight time points during fruit development. This analysis of gene expression lays the groundwork for a molecular understanding of fruit growth and development in apple.

**Results:**

Using ANOVA analysis of the microarray data, 1955 genes showed significant changes in expression over this time course. Expression of genes is coordinated with four major patterns of expression observed: high in floral buds; high during cell division; high when starch levels and cell expansion rates peak; and high during ripening. Functional analysis associated cell cycle genes with early fruit development and three core cell cycle genes are significantly up-regulated in the early stages of fruit development. Starch metabolic genes were associated with changes in starch levels during fruit development. Comparison with microarrays of ethylene-treated apple fruit identified a group of ethylene induced genes also induced in normal fruit ripening. Comparison with fruit development microarrays in tomato has been used to identify 16 genes for which expression patterns are similar in apple and tomato and these genes may play fundamental roles in fruit development. The early phase of cell division and tissue specification that occurs in the first 35 days after pollination has been associated with up-regulation of a cluster of genes that includes core cell cycle genes.

**Conclusion:**

Gene expression in apple fruit is coordinated with specific developmental stages. The array results are reproducible and comparisons with experiments in other species has been used to identify genes that may play a fundamental role in fruit development.

## Background

Fruit-bearing crop species are an important component of the human diet providing nutrition, dietary diversity and pleasure. Fruit are typically considered an enlarged organ that surrounds the developing seeds of a plant, or the ripened ovary of a flower together with any associated accessory parts [1]. The development and final form of the fruiting body is widely varied, ranging from minimally expanded simple dehiscent (non-fleshy) fruit of the model plant Arabidopsis, through expanded ovaries of tomato, to complex fruiting organs with several different expanded tissues, such as found in the pome fruit [1]. Common to all fruit is the developmental process that results in expansion of tissue near the seed in a coordinated manner with seed development (usually, but not always, enclosing the seed). At early stages during development (both before and after successful fertilization, and sometimes in the absence of fertilization) the fruit tissue undergoes several rounds of cell division, followed (usually) by cell expansion during which the fruit stores metabolites and energy, in the form of starch or sugars (e.g. tomato development [2-4]). Subsequently, usually after the seeds mature, the fruit undergoes a series of biochemical changes that convert starches into more available and attractive compounds, such as sugars, as well as producing volatile secondary metabolites that are thought to function as attractants for animals or insects which disperse the seed.

Morphological and physiological studies of fruit have led to considerable understanding of the physical and biochemical events that occur as fruit mature and ripen [1,3,5], however it is only relatively recently that genomic approaches have been used to investigate fruit development [4,6-9]. As a result of excellent genetic resources and the application of molecular and genomic approaches, tomato has become the best studied indehiscent fruit. Domestication of tomatoes has resulted in the increase of fruit size from a few grams to varieties 1000-fold larger [10]. The physiological events leading to the expansion of the ovary wall of the tomato flower and in particular the events that occur around tomato ripening have been well described (for reviews see Gillaspy et al. [2]; Giovanonni [3]). More recently, molecular approaches have been used to study global gene expression in tomato [11-13] allowing identification of large numbers of genes potentially involved in fruit development and ripening.

In other fruit crops, microarrays have been used to examine gene expression during the development and in particular the ripening of fruits such as strawberry [6], peach [14], pear [15], and grape [8,9]. These studies have identified genes involved in fruit flavour and genes associated with distinct stages of fruit development.

Apples (*Malus *× *domestica *Borkh. also known as *M. pumila*) are members of the Rosaceae family, sub family pomoideae, which includes crop species such as pear, rose and quince. Members of the pomoideae have a fruit that consists of two distinct parts: an expanded ovary corresponding to the "core" which is homologous to the tomato fruit; and the cortex or edible portion of the fruit which is derived from the fused base of stamens, petals and sepals [1,16], which expands to surround the ovary. Fruit develop over a period of 150 days from pollination to full tree ripeness with a simple sigmoidal growth curve [17,18]. Physiological studies of apple fruit development have focused on measures of ripeness such as colour changes and breakdown of starch to form the palatable sugars. From such studies, it has been shown that floral buds contain a small amount of starch that is metabolized quickly after pollination. Starch levels then build up in fruit coordinate with cell expansion. At about 100 days after pollination starch levels begin to decline again and fruit sugars increase, until the fruit are fully ripe [19]. Like tomato, apple undergoes an ethylene-dependent ripening stage [20,21] and transgenic apples with reduced ethylene production fail to produce skin colour changes and appear to lack production of volatile compounds typically associated with apples [22].

Apple is functionally a diploid with 2n = 34 and a genome of moderate size (1C = 2.25 pg [23] which corresponds to approximately 1.5 × 10^9 ^bp) making genomic approaches to the study of its biology reasonable. Recently an EST sequencing approach has been used to identify apple genes [24]; unigenes derived from this sequencing project were used to design the oligonucleotides used in this work. Two groups have published apple microarray analyses [22,25]. Lee et al. [25] used a 3484 feature cDNA array to identify 192 apple cDNAs for which expression changes during early fruit development. Using the same ~13000 gene (15726 feature) apple oligonucleotide array described in this paper, Schaffer et al. [22] identified 944 genes in fruit that respond to ethylene treatment and associated changes in gene expression with changes in fruit volatiles.

In the work described in this paper, microarrays have been used to study the developmental processes occurring during fruit formation from pollination to full tree ripeness. In pome fruit both core (ovary) and cortex (hypanthium) tissues expand. Understanding the regulation of the events required to produce a complex apple fruit, including the division and expansion of cells from different floral structures is the ultimate aim of this work. Using microarrays we show that large groups of genes are co-ordinately expressed at specific stages of fruit development. We have identified cell division genes for which expression coincides with the period of cell division in apple fruit and have identified starch metabolic enzymes likely to be involved as fruit store and then metabolize starch. Using a comparative approach we have identified a number of genes for which expression patterns are similar in both apple and tomato fruit development and may be involved in similar fundamental processes in fruit development.

## Results

### Microarray analysis of apple fruit development

**Figure 1:**
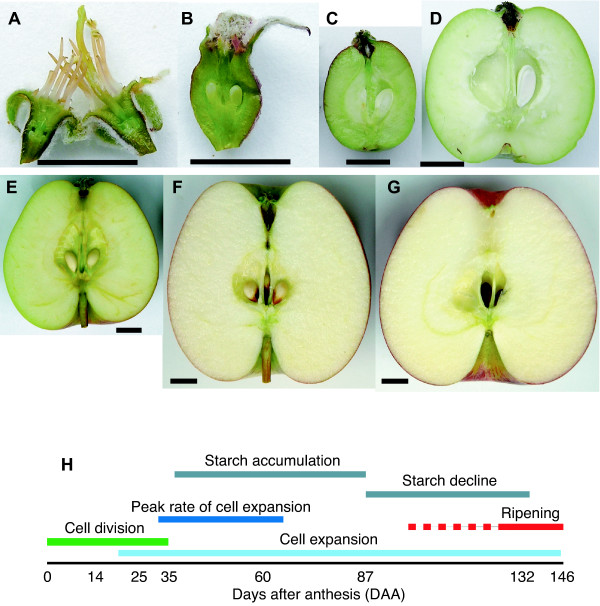
**Apple fruit development**. Apple fruit at various stages of development. A, 0 DAA, B, 14 DAA, C, 35 DAA, D, 60 DAA, E, 87 DAA, F, 132 DAA, G, 146 DAA. H, diagram of fruit development showing the timing of major physiological events and the sampling time points, adapted from [17–19]. Ripening is shown as a solid and dashed red, solid from the time of the climacteric and dashed for events prior to the climacteric. Bar = 1 cm.

When apple trees (*Malus domestica *'Royal Gala') were at full bloom (greater than 50% of buds open) individual fully open flowers were tagged and trees separated into two biological replicates (Rep1 and Rep2). Based on physiological and morphological studies of apple fruit development [17,19] eight time points were selected for sampling (Figure 1). The first sample 0 Days After Anthesis (DAA) was taken at the same time that fully open flowers were tagged. The 14 and 25 DAA sampling time points coincide with the period of cell division that occurs after pollination. At 35 DAA cell division has ceased, the rate of cell expansion increases and starch accumulation begins. 60 DAA coincides with the greatest rate of cell expansion and starch accumulation. By 87 DAA the rate of cell expansion has declined but cell expansion continues at a reduced rate until full ripeness, starch levels peak shortly after this timepoint. In the year in which the samples were taken harvest ripeness was at 132 DAA, at this stage starch levels are rapidly declining and fruit sugars increasing, skin colour is still changing and while some flavour compounds are present full "apple flavour" has not yet developed. By 146 DAA fruit were "tree ripe" at this stage fruit have strong colour and have fully developed flavour, almost all the starch present has been converted into fruit sugars and some flesh softening has occurred. While developmental events that occur prior to full bloom are significant in the developmental program leading to the final fruit, samples prior to full bloom were not considered in this work. RNA was extracted from samples from both replicates, labelled and hybridized to an array of 15726 oligonucleotides (45–55 bases long) designed from 15145 unigenes representing approximately 13000 genes. All samples were compared (using a dye swap design) to genomic DNA (gDNA) as a common reference, making samples directly comparable, the absolute expression of all the samples is shown in Additional file 1.

### Four major groups of co-ordinately expressed genes during fruit development

**Figure 2:**
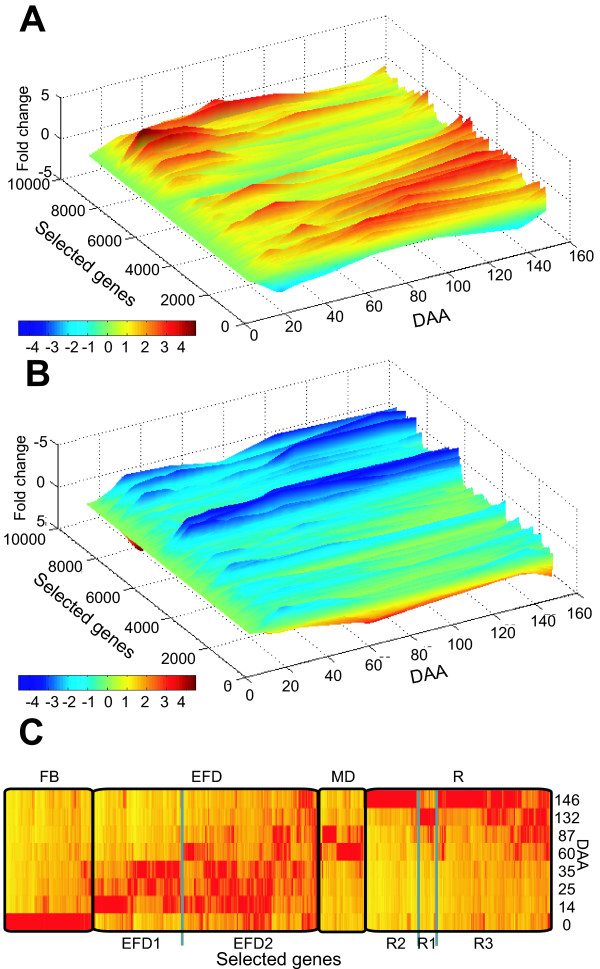
**Clustering of genes changing during fruit development**. Cluster analysis of gene expression. A and B, Expression patterns for the whole array were clustered and then plotted in 3-D space (MATLAB, version 6.0; The Mathworks). Genes with no expression changes or with greater than 5 fold changes were excluded, leaving 8719 genes. y-axis shows fold change. C, The 1955 developmentally regulated genes selected by ANOVA (FDR = 0.01) were clustered by their geometric means. Vertical lines represent transcript level observed for each EST from 0 to 146 DAA, minimum expression (yellow), maximum (red). Major clusters are: floral bud or full bloom (FB); early fruit development (EFD); mid-development (MD); and ripening (R). The EFD and R clusters were further sub-clustered and indicated by EFD1, EFD2, R1, R2 and R3.

To examine global changes in gene expression, 8719 genes which changed in expression during fruit development (genes with greater than 5-fold change were excluded in order to see the pattern from genes exhibiting smaller changes, inclusion of these genes did not alter the pattern of expression seen for the majority of genes) were grouped using hierarchical clustering and visualized by plotting expression in 3-dimensional space (Figure 2A and 2B). This global analysis of the microarray shows four major patterns of coordinated gene expression. A group of genes was identified with expression in floral buds but are down-regulated throughout fruit development, a second group of genes was up-regulated early in development and down-regulated later, two additional groups of genes were up-regulated during the middle stages of development and during ripening. By contrast with the results seen for tomato [13], there was no sharp change in global expression patterns at ripening, but this difference is likely to reflect differences in sampling.

To identify those genes that changed expression significantly, a one way ANOVA (model y = time) was applied to the entire dataset. Using a non-adaptive false discovery rate (FDR) control [26] of 0.01, 1986 features were identified (corresponding to 1955 genes) where gene expression changed significantly during fruit development. Hierarchical clustering identified four groups of genes with similar patterns of expression during fruit development (Figure 2C, and Additional file 1, which lists the entire dataset). The full bloom (FB) cluster contained 314 genes (315 features) with high expression at 0 DAA and then low expression during the rest of fruit development. The early fruit development (EFD) cluster contained 814 genes (819 features) where expression peaked between 14 and 35 DAA. The EFD cluster consisted of two weaker sub-clusters: EFD1, a group of 320 genes (326 features) which had high expression early and then very low expression later in development; and EFD2 a group of 493 genes (493 features) with high expression early and moderate expression later in development. The mid development cluster (MD) contained 168 genes (169 features) with expression peaking at 60 and 87 DAA and low expression at other stages of development. The ripening cluster (R) contains 668 genes (681 features) with expression low initially and eventually peaking late in fruit development. The R cluster could be clustered into three further sub-clusters: R1 70 genes (70 features) where expression peaked at harvest ripe (132 DAA) and was low at other stages of development; R2 191 genes (195 features) where expression was very low throughout development until tree ripe (146 DAA); and R3 406 genes (408 features) where expression peaked at tree ripe (146 DAA) but some expression was present at earlier stages of development. Both approaches to clustering identified four major groups of co-ordinately expressed genes suggesting these correspond to major phases of fruit development.

### Validation of microarray expression by quantitative RT-PCR

**Figure 3:**
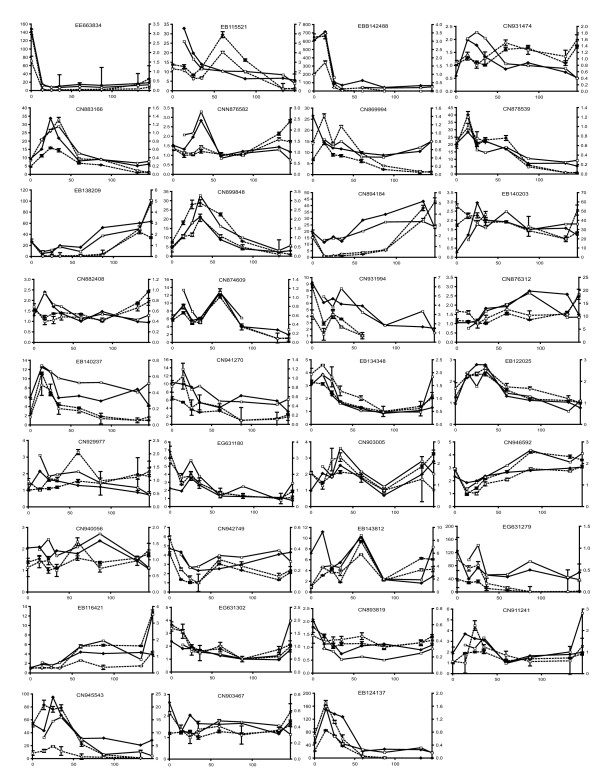
**Validation of array expression patterns**. The pattern of expression for a selection of ESTs was confirmed by quantitative RT-PCR using primers designed close to the array oligo. Graphs show transcript levels from the array (solid lines) for Rep1 (filled diamonds) and Rep2 (open squares) compared with transcript levels from qRT-PCR (dashed lines, mean and standard error for each sample) for Rep1 (filled diamonds) and Rep2 (open squares). X axes show DAA, the left Y axes show relative qRT-PCR expression, the right Y axes show absolute array expression. The genbank accession is shown for each EST.

To examine the reliability of gene expression patterns identified from the microarray we used quantitative reverse transcriptase-PCR (qRT-PCR) to examine steady-state RNA levels during fruit development. Genes for qRT-PCR were initially selected from the list of genes that significantly changed their expression during fruit development. The list of regulated genes was ordered from most significant to least significant and genes for qRT-PCR selected at regular intervals from this list (approximately every 50^th ^gene). Several genes were also chosen for qRT-PCR to confirm expression patterns of genes in particular pathways (see below). Three housekeeping genes were used to normalize qRT-PCR results: an actin gene (Genbank accession CN927806); a GAPDH gene (Genbank accession CN929227) and a gene of unknown function which was selected on the basis of low variability in microarray experiments (Genbank accession CN908822). qRT-PCR expression profiles were compared with microarray expression profiles (Figure 3) and scored as matching if they agreed at all developmental stages or if the majority of stages were in agreement and the significant changes in expression also agreed. By these criteria 74% (26 out of 35) of genes had the same pattern of expression in the microarray experiment as in the qRT-PCR experiment. Interestingly no relationship was observed between the reproducibility of the expression pattern and the significance of the microarray data as determined by ANOVA.

### Genes in different functional classes are expressed at different times during fruit development

To examine the changes in gene function that were occurring during fruit development, functional classes for the apple genes were identified using the Arabidopsis protein function classification defined by the Munich Information center for Protein Sequences (MIPS, using the funcat-1.3 scheme [27]). For all the apple genes represented on the array, the Arabidopsis gene with the best sequence similarity based on BLAST analysis was selected [28], with a threshold expect value of 1 × e^-5^, and MIPS functional categories for that Arabidopsis gene assigned to the apple gene. This relatively non-stringent threshold was chosen in order to obtain functional classifications for the majority of apple genes on the array. Table 1 shows the number of apple genes, the number of genes with Arabidopsis matches, the number of matches to unique Arabidopsis genes and the number of MIPS functional categories for the entire array, for the 1986 features selected as changing during fruit development, and for the clusters and sub-clusters.

**Table 1:** Distribution of array features

Subset/cluster^a^	ESTs^b^	Apple genes^c^	Apple genes with hit to Arabidopsis^d^	Unique Arabidopsis genes^e^	Functional categories^f^
whole array	15726	15145	11949	8256	63732
Selected 1986	1983	1955	1442	1330	7523
FB	315	314	225	212	1141
EFD	819	812	603	566	3042
MD	169	168	126	124	653
R	681	668	495	474	2722
EFD1	326	320	236	220	1128
EFD2	493	493	368	356	1916
R1	70	70	54	53	300
R2	195	191	154	154	885
R3	408	406	284	277	1552

The table shows the number of genes on the whole array and within the clusters as well as the number of Arabidopsis homologues and the number of MIPS function classifications identified.

a FB = full bloom; EFD = Early fruit development; MD = Mid-development; R = ripening; R1, R2, R3 = Ripening subclusters 1, 2 and 3; EFD1, EFD2 = early fruit development subclusters 1 and 2.

b The number of apple ESTs represented by the features on the array.

c The number of apple genes, tentative contigs or singletons identified by the ESTs on the array.

d Apple genes were compared with the Arabidopsis predicted protein set using BLASTx to identify similar Arabidopsis genes, the best match (with expect value better than 1 × e^-5^) was used for subsequent functional analysis.

e The number of unique Arabidopsis genes identified by BLASTx using the apple genes, in many cases multiple apple genes had strongest similarity to the same Arabidopsis gene, thus fewer Arabidopsis genes were identified than apple genes.

f Functional categories found for the Arabidopsis genes were identified using the MIPS dataset funcat 1.3.

The distribution of functional categories for the entire array is shown in Table 2 and compared with the distribution of the 1955 genes selected as changing significantly during fruit development, the major clusters and the sub-clusters. The distribution of MIPS functional categories changes between the whole array and the genes selected as changing during fruit development suggest that the genes selected are not a random selection from the array as a whole. For example, there appears to be a higher representation of genes associated with metabolism in the fruit development genes (20.3% vs 16.1% for the whole array) suggesting developing fruit are more active metabolically. Interestingly, there is a slight increase in the unclassified category in the selected fruit development genes 16.7% vs 15.7% for the whole array, while in the ripening cluster the unclassified category is under-represented compared to other clusters (15.2% vs 17.4 to 17.8%), which may reflect the amount of research focused on identifying and characterizing genes involved in the late stages of ripening as compared with early events in fruit development.

**Table 2:** Functional classification

	Mips code^a^	Whole array^b^	selected	FB	EFD	MD	R	EFD1	EFD2	R1	R2	R3
Metabolism	1	16.1	20.3	21.5	20.1	17.2	20.9	18.3	21.1	21.7	25.4	18.4
Energy	2	2.9	3.4	3.0	2.8	2.1	4.5	2.2	3.1	3.0	5.0	4.4
Cell Cycle and DNA processing	3	2.9	2.5	1.8	3.4	1.4	1.9	3.3	3.5	0.7	1.9	2.4
Transcription	4	5.2	4.1	4.3	4.1	4.1	3.9	4.4	4.0	3.3	3.1	4.6
Protein synthesis	5	2.0	1.7	1.5	1.6	1.8	2.0	1.5	1.6	2.7	0.7	2.6
Protein fate	6	6.6	5.4	4.6	5.0	5.5	6.0	4.5	5.3	4.0	4.7	7.1
Cellular transport & mechanisms	8	2.4	1.7	1.8	1.6	2.6	1.7	2.1	1.3	0.7	1.7	1.8
Cellular comm/signaling	10	6.4	5.6	6.5	5.5	5.1	5.6	5.9	5.4	9.0	5.6	4.9
Cell rescue, defense & virulence	11	3.6	4.0	4.1	4.1	4.6	3.6	3.3	4.6	5.7	4.3	2.9
Regulation of/interaction with cellular environment	13	1.7	1.6	2.1	1.7	2.8	1.1	1.7	1.7	0.3	1.1	1.3
Cell fate	14	3.2	2.6	2.3	2.5	1.5	3.2	2.4	2.6	3.7	2.6	3.3
Systemic regulation of/interaction with environment	20	1.1	1.3	1.5	1.3	1.1	1.1	1.9	0.9	1.3	1.1	1.1
Development	25	1.0	1.2	1.2	1.4	1.1	0.9	1.2	1.5	2.0	0.6	0.8
Transposable elements, viral and plasmid proteins	29	0.1	0.1	0.1	0.0	0.0	0.1	0.0	0.1	0.0	0.2	0.0
Control of cellular organisation	30	2.7	3.2	2.7	3.8	4.6	2.4	4.8	3.3	2.7	2.3	2.4
Subcellular localisation	40	19.1	18.1	15.9	17.4	19.8	19.2	17.5	17.4	16.0	18.0	20.5
Protein activity regulation	62	0.0	0.1	0.2	0.1	0.0	0.0	0.2	0.1	0.0	0.0	0.0
Protein with binding function or cofactor requirement	63	3.2	2.9	2.7	2.8	4.4	2.8	2.3	3.1	2.3	3.1	2.7
Storage protein	65	0.1	0.0	0.1	0.0	0.0	0.0	0.1	0.0	0.0	0.1	0.0
Transport facilitation	67	3.9	3.6	4.4	3.2	2.9	3.7	3.4	3.1	3.0	4.3	3.7
Unclassified	98 or 99	15.7	16.7	17.8	17.4	17.5	15.2	19.1	16.4	18.0	14.2	15.1

The table shows the distribution of classifications as a percentage of the total number of classifications.

a Apple genes for each EST on the array were used to identify Arabidopsis homologues using BLAST with a cutoff of 1 e^-5^. Where a putative homologue was identified, the Arabidopsis MIPS (Munich Information centre for Protein Sequences, funcat version 1.3) classification(s) for that gene were applied to the apple EST.

b For the whole array, for the features selected as changing during fruit development, and for each of the clusters and sub-clusters the frequency of occurrence for each functional category is shown as a percentage of the total number of functional categories for that cluster (or sub-cluster). FB = Full bloom; EFD = early fruit development; MD = mid-development; R = ripening; R1, R2, R3 = the 3 ripening sub-clusters; EFD1, EFD2 = the 2 early fruit development sub-clusters.

Within the four major clusters, the genes with peak expression in mid-development have a reduced representation of genes associated with metabolism (17.2% vs 20.1 to 21.5%) suggesting this stage of fruit development might be less metabolically active or use fewer different metabolic genes. In contrast, cellular transport and transport mechanism functions are more highly represented in the mid-development cluster (2.6% vs 1.6 to 1.8%) at the time when fruit are taking up nutrients and water most rapidly.

Control of cellular organization functions are represented more in the EFD and MD clusters (3.8% and 4.6% vs FB2.7% and R2.4%) consistent with this period being a stage of fruit development where the structure of the fruit cells is changing rapidly. In the ripening cluster there is an over-representation of genes in the "energy" category (4.5%) with the lowest representation in mid-development (2.1%). In addition the R2 (peak expression at tree ripe) sub-cluster is over-represented (compared with the other ripening sub-clusters, R1 and R3) in the "metabolism" category (25.4% vs 21.7 and 18.4%) correlating with changes in energy and metabolism during late ripening.

One feature of note was the higher proportion of genes with a cell cycle classification in the EFD cluster (FB 1.8%, EFD 3.4%, MD 1.4%, R 1.9%). The EFD cluster contains genes for which expression peaks in the first 30 days of fruit development, the stage of development when cells are dividing [17,18]. This developmental period involves the division of specific cells to form the final apple fruit shape and since there appeared to be an increase in cell cycle associated genes during this period we identified the genes associated with the cell cycle classification for each cluster (FB 17 genes, EFD 61 genes, MD 8 genes, R 42 genes) and their annotations (Table 3). These lists are likely to include those genes important in the regulation of fruit size and shape. For example, analysis of these lists identified three core cell cycle genes (see below), which will be the focus of future research.

**Table 3:** Annotation of cell cycle genes by cluster

**FB cluster**				
EST	Genbank acc.	Best A. thaliana hit^a^	e value	Description^b^

5019	CN936403	AT5G44680.1	1e-40	methyladenine glycosylase family protein
5126	EB107042	AT2G38620.1	9e-80	CDKB1;2 cell division control protein
33679	CN929052	AT2G47420.1	9e-18	dimethyladenosine transferase
59120	CN862228	AT5G42320.1	2e-12	zinc carboxypeptidase family protein
67405	CN864463	AT5G53000.1	3e-31	protein phosphatase 2A-associated 46 kDa protein
86932	EB119954	AT1G01490.1	2e-19	heavy-metal-associated domain-containing protein
124169	CN937737	AT1G18660.1	3e-67	zinc finger (C3HC4-type RING finger) family protein
134415	CN888558	AT3G62600.1	1e-153	DNAJ heat shock family protein
140667	CN938500	AT2G24490.1	8e-46	replication protein, putative
222173	CN876164	AT4G11010.1	9e-47	nucleoside diphosphate kinase 3, mitochondrial (NDK3)
226032	EG631233	AT3G08500.1	3e-48	myb family transcription factor (MYB83)
254247	CN912925	AT1G10290.1	3e-49	dynamin-like protein 6 (ADL6)
256645	EB151655	AT1G79350.1	1e-77	EMB1135 DNA-binding protein, putative
257305	CN908171	AT3G57550.1	3e-41	guanylate kinase 2 (GK-2)
258270	CN914773	AT2G30110.1	1e-179	ubiquitin activating enzyme 1 (UBA1)
264677	CN910366	AT3G48160.2	6e-68	E2F-like repressor E2L3 (E2L3)
264992	CN917058	AT5G23430.1	1e-53	transducin family protein/WD-40 repeat family protein

**EFD cluster**				

EST	Genbank acc.	Best A. thaliana hit	e value	Description

12163	EB109178	AT3G28030.1	2e-27	UV hypersensitive protein (UVH3)
14094	CN931474	AT2G01440.1	6e-15	ATP-dependent DNA helicase, putative
15274	CN932236	AT3G25500.1	8e-26	FH2 domain-containing protein
19893	CN925129	AT1G73540.1	3e-11	ATNUDT21 MutT/nudix family protein
29516	EB111254	AT2G39730.1	9e-72	RuBisCO activase
31066	CN927871	AT3G23890.1	8e-13	DNA topoisomerase II
33027	CN928590	AT3G25500.1	3e-39	FH2 domain-containing protein
43417	EB113579	AT1G69770.1	3e-06	chromomethylase 3 (CMT3)
45185	CN857495	AT5G05510.1	2e-25	low similarity to SP:O60566 Mitotic checkpoint serine/threonine-protein kinase BUB1 β
62518	EB116342	AT3G08910.1	7e-67	DNAJ heat shock protein
64262	CN850169	AT2G30200.1	1e-148	T27E13_6
85474	CN869267	AT1G68760.1	6e-54	ATNUDT1 MutT/nudix family protein
91885	CN871666	AT1G10520.1	3e-15	DNA polymerase lambda (POLL)
93419	CN874495	AT5G26751.1	4e-58	shaggy-related protein kinase α/ASK-α (ASK1)
95093	CN875141	AT5G18110.1	5e-60	novel cap-binding protein (nCBP)
105540	CN886787	AT3G51770.1	1e-111	similar to tetratricopeptide repeat (TPR)-containing protein
111728	EB124553	AT1G44900.1	3e-50	DNA replication licensing factor
118006	EB125634	AT2G21790.1	8e-45	ribonucleoside-diphosphate reductase small chain, putative
119405	CN887179	AT1G68010.1	1e-81	glycerate dehydrogenase/NADH-dependent hydroxypyruvate reductase
120390	CN890521	AT1G21660.1	7e-12	low similarity to SP:O14976 Cyclin G-associated kinase
138266	CN937814	AT2G17120.1	3e-79	peptidoglycan-binding LysM domain-containing protein
142020	CN939277	AT2G38810.1	2e-48	histone H2A, putative
142920	EB127800	AT5G57850.1	2e-08	aminotransferase class IV family protein
148629	EB138792	AT3G22630.1	2e-36	20S proteasome β subunit D (PBD1) (PRGB)
149453	CN897394	AT5G55230.1	1e-118	ATMAP65-1 Binds and bundles microtubules
149668	CN897544	AT4G36080.1	1e-103	FAT domain-containing protein/phosphatidylinositol 3- and 4-kinase family protein
151134	EB139596	AT2G42580.1	5e-24	tetratricopeptide repeat (TPR)-containing protein
151602	CN898773	AT5G13780.1	8e-81	GCN5-related N-acetyltransferase, putative, similar to ARD1 subunit
152213	CN940414	AT2G35040.1	1e-112	AICARFT/IMPCHase bienzyme family protein
153604	EB140203	AT1G55350.1	0	EMB1275 calpain-type cysteine protease family
153992	CN900578	AT2G21790.1	1e-160	R1 ribonucleoside-diphosphate reductase small chain, putative
155385	CN901052	AT2G21790.1	2e-83	R1 ribonucleoside-diphosphate reductase small chain, putative
155966	CN901211	AT5G61060.1	2e-34	histone deacetylase family protein
159200	CN940759	AT2G14880.1	6e-36	SWIB complex BAF60b domain-containing protein
162529	CN942994	AT3G44110.1	1e-152	DNAJ heat shock protein, putative (J3)
163128	CN943384	AT1G20930.1	1e-102	CDKB2;2 cell division control protein, putative
163154	CN943405	AT5G61060.1	2e-84	histone deacetylase family protein
166835	EE663942	AT3G17880.1	1e-58	tetratricoredoxin (TDX)
170408	EB140959	AT3G08910.1	7e-59	DNAJ heat shock protein, putative
170963	CN882668	AT2G46225.1	2e-20	ABI1L1 Encodes a subunit of the WAVE complex
171493	CN883039	AT2G29570.1	1e-111	PCNA2 proliferating cell nuclear antigen 2 (PCNA2)
172325	CN883596	AT5G08020.1	7e-91	similar to replication protein A1 (Oryza sativa)
173799	EB141951	AT2G27960.1	6e-37	CKS1 cyclin-dependent kinase
180731	CN904791	AT1G75690.1	2e-55	chaperone protein dnaJ-related
181072	CN904980	AT3G18190.1	0	chaperonin, putative
184975	EB148197	AT5G44680.1	1e-90	methyladenine glycosylase family protein
186444	EB149644	AT3G19420.1	2e-12	MLD14.22
186960	EB150084	AT3G08690.1	9e-27	ubiquitin-conjugating enzyme 11 (UBC11), E2
213416	EB157314	AT1G62990.1	1e-126	homeodomain transcription factor (KNAT7)
220588	EB132350	AT3G48590.1	2e-15	CCAAT-box binding transcription factor Hap5a, putative
220604	CN948726	AT4G33260.1	8e-17	WD-40 repeat family protein
245977	CN903005	AT3G26730.1	1e-49	zinc finger (C3HC4-type RING finger) family protein
256235	CN913864	AT2G31320.1	0	NAD(+) ADP-ribosyltransferase, putative
256449	CN916743	AT3G22890.1	1e-165	sulfate adenylyltransferase 1/ATP-sulfurylase 1 (APS1)
257853	CN914478	AT5G52640.1	0	heat shock protein 81-1 (HSP81-1)
261756	CN908391	AT2G25050.1	5e-07	formin homology 2 domain-containing protein
264654	CN910347	AT5G67100.1	5e-87	DNA-directed DNA polymerase α catalytic subunit, putative
265667	CN910570	AT5G16270.1	3e-06	Rad21/Rec8-like family protein
266414	EB152178	AT5G40010.1	1e-112	AAA-type ATPase family protein
315707	CN915704	AT1G03080.1	4e-25	kinase interacting family protein
318786	CN949202	AT1G04820.1	4e-63	tubulin α-2/α-4 chain (TUA4)

**Mid dev cluster**				

EST	Genbank acc.	Best A. thaliana hit	e value	Description

109011	CN880656	AT1G29400.1	4e-77	RNA recognition motif (RRM)-containing protein
144884	CN894104	AT1G03190.1	1e-33	DNA repair protein/transcription factor protein (UVH6)
146572	CN895134	AT2G15580.1	2e-14	zinc finger (C3HC4-type RING finger) family protein
167024	EG631355	AT5G66770.1	0	scarecrow transcription factor family protein
182020	EB143575	AT1G69840.1	3e-73	band 7 family protein
185452	EB148668	AT1G07350.1	1e-31	transformer serine/arginine-rich ribonucleoprotein, putative
214774	CN946063	AT1G26830.1	1e-75	CUL3 Cullin, putative, similar to Cullin homolog 3 (CUL-3)
268033	CN918413	AT5G64610.1	1e-142	histone acetyltransferase, putative

**Ripening cluster**				

EST	Genbank acc.	Best A. thaliana hit	e value	Description

541	CN934040	AT3G57220.1	1e-113	UDP-GlcNAc:dolichol phosphate N-acetylglucosamine-1-phosphate transferase, putative,
11629	EB109003	AT1G34260.1	1e-07	phosphatidylinositol-4-phosphate 5-kinase family protein
15678	CN932487	AT5G51600.1	3e-85	microtubule associated protein (MAP65/ASE1) family protein
57477	CN860296	AT2G44270.1	1e-164	contains Pfam profile PF01171: PP-loop family
59442	CN862410	AT1G73460.1	1e-35	protein kinase family protein Pfam:PF00069
64262	CN850169	AT2G30200.1	1e-148	expressed protein T27E13_6
64821	CN863160	AT5G51570.1	1e-141	band 7 family protein
68274	CN864737	AT5G26940.1	3e-59	exonuclease family protein
89547	CN873630	AT3G61140.1	2e-09	COP9 signalosome complex subunit 1/CSN complex subunit 1
89732	EB121320	AT4G12600.1	8e-18	ribosomal protein L7Ae/L30e/S12e/Gadd45 family protein
93568	CN874587	AT3G10940.1	1e-108	similar to protein phosphatase PTPKIS1 protein
107778	CN871562	AT1G77600.1	6e-07	expressed protein, weak similarity to Pds5
111901	CN879476	AT1G14400.1	1e-39	ubiquitin-conjugating enzyme 1 (UBC1), E2
130406	CN891639	AT3G27180.1	5e-08	expressed protein MYF5.5
132758	CN892125	AT5G48330.1	9e-55	regulator of chromosome condensation (RCC1) family protein
134470	CN888599	AT2G29900.1	2e-35	presenilin family protein
141926	CN939221	AT5G50960.1	1e-163	similar to Nucleotide-binding protein 1 (NBP 1)
143463	CN890171	AT1G69670.1	9e-75	ATCUL3B cullin, putative
146658	CN895184	AT5G12200.1	0	dihydropyrimidinase (PYD2)
147359	EB138102	AT1G05910.1	1e-111	cell division cycle protein 48-related/CDC48-related
147418	CN895629	AT3G18600.1	4e-32	DEAD/DEAH box helicase, putative
150678	CN898212	AT3G07760.1	3e-28	expressed protein MLP3.21
155382	CN901049	AT3G24320.1	3e-73	DNA mismatch repair MutS family (MSH1)
159868	EB128540	AT2G19770.1	5e-45	profilin 4 (PRO4) (PFN4)
172304	CN883582	AT3G48530.1	2e-72	CBS domain-containing protein
175286	CN904072	AT4G25130.1	1e-100	peptide methionine sulfoxide reductase, putative
184340	EB147575	AT3G13230.1	2e-77	expressed protein MDC11.5
185727	EB148939	AT5G21990.1	1e-107	tetratricopeptide repeat (TPR)-containing protein
186037	EB149246	AT4G25130.1	3e-71	peptide methionine sulfoxide reductase, putative
216840	CN947326	AT4G04955.1	3e-45	ATALN Encodes an allantoinase
219785	CN851874	AT2G30200.1	1e-148	expressed protein T27E13_6
221777	CN875931	AT5G17570.1	1e-115	tatD-related deoxyribonuclease family protein
221885	EB122552	AT1G55860.1	2e-19	ubiquitin-protein ligase 1 (UPL1)
225203	CN877466	AT1G68370.1	9e-74	gravity-responsive protein (ARG1)
228881	CN878128	AT1G77930.1	1e-105	DNAJ heat shock N-terminal domain-containing protein
229438	CN878271	AT1G20760.1	2e-30	calcium-binding EF hand family protein
229922	CN878558	AT1G20110.1	4e-73	zinc finger (FYVE type) family protein
257846	CN914471	AT1G15240.1	8e-26	phox (PX) domain-containing protein
266842	CN916307	AT2G45620.1	4e-09	nucleotidyltransferase family protein
267005	CN916212	AT4G28000.1	7e-51	AAA-type ATPase family protein
267748	CN918233	AT5G41370.1	4e-13	XPB1 involved in both DNA repair and transcription
289972	CN884487	AT3G23610.1	5e-60	dual specificity protein phosphatase (DsPTP1)

a ESTs that change during fruit development were used to identify apple genes and the best Arabidopsis homolog (by BLAST) was found for that apple gene. Where a sequence similarity was better than 1 × e-5 the MIPS functional category for that Arabidopsis gene was determined.

b Genes with the functional category "Cell cycle and DNA processing" were identified in each array cluster and ESTs in those clusters and the annotation of the Arabidopsis homolog is shown.

### Expression of core cell cycle genes

From morphological studies apple fruit cells go through at least four rounds of cell division during the first 30 days after pollination with total cell number increasing 10 fold [17,18]. At around 30 DAA the cells that make up the core and cortex of the mature fruit stop dividing and the rate of cell expansion increases. The control of cell division and cell expansion is a key part of the developmental regulation of fruit and is likely to affect final fruit size as well as texture and the balance between tissue types.

Using an analysis of the Arabidopsis genome sequence, Vanderpoele et al. [29] identified 61 core cell cycle genes; this list has been expanded to 88 genes, including several previously unrecognized groups [30]. Expression analysis in Arabidopsis has demonstrated that many of these core cell cycle genes have regulated steady state RNA levels [30]. To determine if any of these core cell cycle genes were regulated in fruit development, we identified apple homologues and examined their expression. As fruit samples were pooled from multiple fruit and because within a fruit cell division is unlikely to be synchronized, we would not expect to be able to detect variation of expression during the cell cycle. However any core cell cycle gene that varied developmentally might be associated with the control of cell division rates during fruit formation and development.

**Figure 4:**
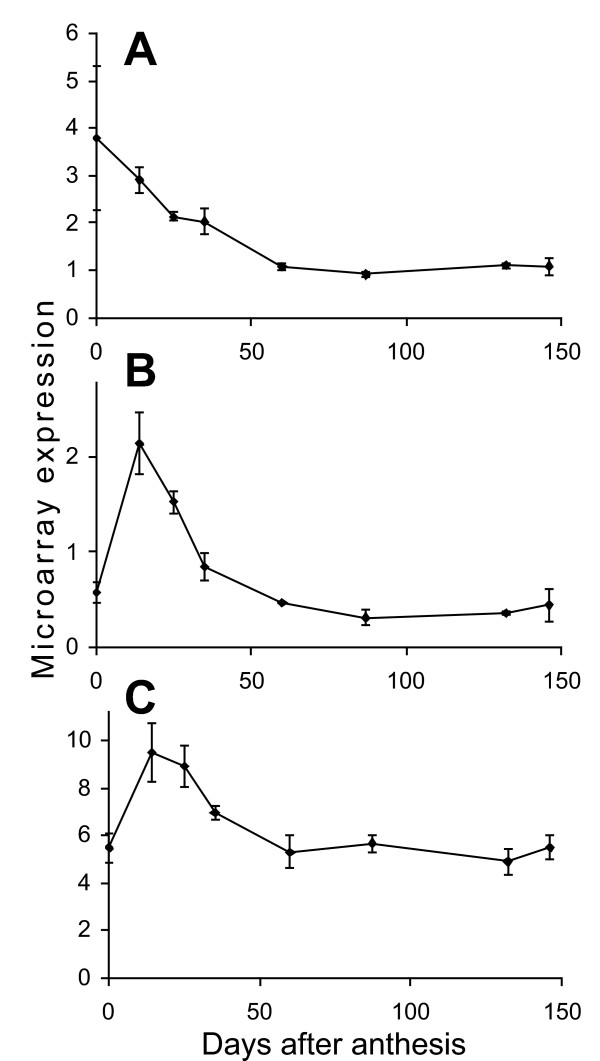
**Expression of core cell cycle genes**. Array expression levels are shown for the three core cell cycle genes that changed significantly during apple fruit development. A, EB107042 a CDKB1;2 homologue, B, CN943384 a CDKB2;2 homologue, C, EB141951 a CKS1 homologue.

Thirty-eight apple genes represented on the apple array have strong sequence similarity to the 88 Arabidopsis cell cycle genes identified by Menges et al. [30], using BLASTx and manual examination of protein sequence alignments (31 have expect value of 1 × e^-40 ^or better). Of these 38 apple genes, only three were in the 1955 genes selected by ANOVA as changing significantly during fruit development (Figure 4). ESTs 5126 (Genbank acc. EB107042), 163128 (Genbank acc. CN943384) and 173799 (Genbank acc. EB141951) all had high levels of expression early in development which declined to relatively low levels after 35 DAA. The three genes have sequence similarity to the Arabidopsis genes At2g38620.1, At1g20930.1 and At2g27960 (expect values of 1 × e^-146^, 1 × e^-150 ^and 6 × e^-37^, respectively). At2G38620.1 is a CDKB1;2 homologue, At1G20930.1 is a CDKB2;2 homologue and At2g27960 is a CKS1 homologue, the two CDKB genes play roles in progression of the cell cycle and the CKS gene is a mitosis specific scaffold protein. At this level of sequence similarity it is not possible to determine if the apple genes represent orthologues of these genes, although similarity of function is likely.

### Expression of genes associated with starch metabolism

Starch metabolism in apple fruit is a physiological process with a well-defined developmental pattern [19]. However, the mechanism by which starch levels are regulated in plants is complex and little is known about how the activity and turnover of starch synthesis and degradation enzymes are mediated in storage tissues such as fruits (reviewed by Smith et al. [31]). To investigate whether there is some regulation of starch metabolic enzymes at the level of transcription in apple fruit, we examined the patterns of expression for several enzymes involved in starch metabolism. Arabidopsis enzymes involved in starch turnover were identified from the starch and sucrose metabolic pathway in the Kyoto Encyclopedia of Genes and Genomes (KEGG) database [32]. Apple genes with significant sequence similarity to the Arabidopsis starch turnover genes (BLAST significance better than 1 × e^-100^) were included in the analysis (Table 4).

**Table 4:** Enzymes involved in Starch metabolism

Enzyme	EC #	A. thaliana gene	Genbank acc.^a^	expect value^b^	qPCR vs array^c^	Localisation
Sucrose synthase	2.4.1.13	At3g43190	EB144194	0	+	plastidic
		At4g02280	CN897963	0	++	unknown
		At5g20830				
		At5g37180				
		At5g49190				

UDP-glucose pyrophosphorylase	2.7.7.9	At5g17310	EG631379	1e-173	+++	endomembrane system

Starch synthase	2.4.1.21	At1g32900	EE663720	0	-	plastidic
		At3g01180	EB121923	0	+++	plastidic

ADP-glucose phosphorylase	2.7.7.27	At1g27680	CN884033	1e-167	+++	plastidic
		At2g21590				
		At4g39210				
		At5g19220				
		At5g48300				
		At1g05610				

Starch phosphorylase	2.4.1.1	At3g29320	EE663644	0	-	plastidic
		At3g46970	EB108842	1e-115	-	unknown

Sucrose-phosphate synthase	2.4.1.14	At5g20280	EB112628	0	++	unknown
		At1g04920	EB123469	0	++	unknown
		At5g11110				
		At4g10120				

β-amylase	3.2.1.2	At4g15210	EB114557	1e-116	+++	plastidic
		At4g17090	EG631202	1e-104	-	plastidic

α-glucosidase	3.2.1.20	At3g45940	EE663791	0	+++	endomembrane system
		At5g11720	EE663790	0	-	endomembrane system
		At5g63840				

Sucrose phosphatase	3.1.3.24	At2g35840	EB156512	0	+++	cytoplasm

Starch metabolism genes were identified and the expression of putative apple starch metabolism genes confirmed by qRT-PCR.

a The representative EST on the array is shown for the best apple gene match to the Arabidopsis gene.

b The significance of the BLAST comparison between the Arabidopsis gene and the best apple gene.

c The degree of correspondence between pattern of gene expression by microarray and the pattern by qPCR. - = no correspondence; + = more than two points of divergence; ++ = good correspondence but some differences; +++ = strong correspondence

**Figure 5:**
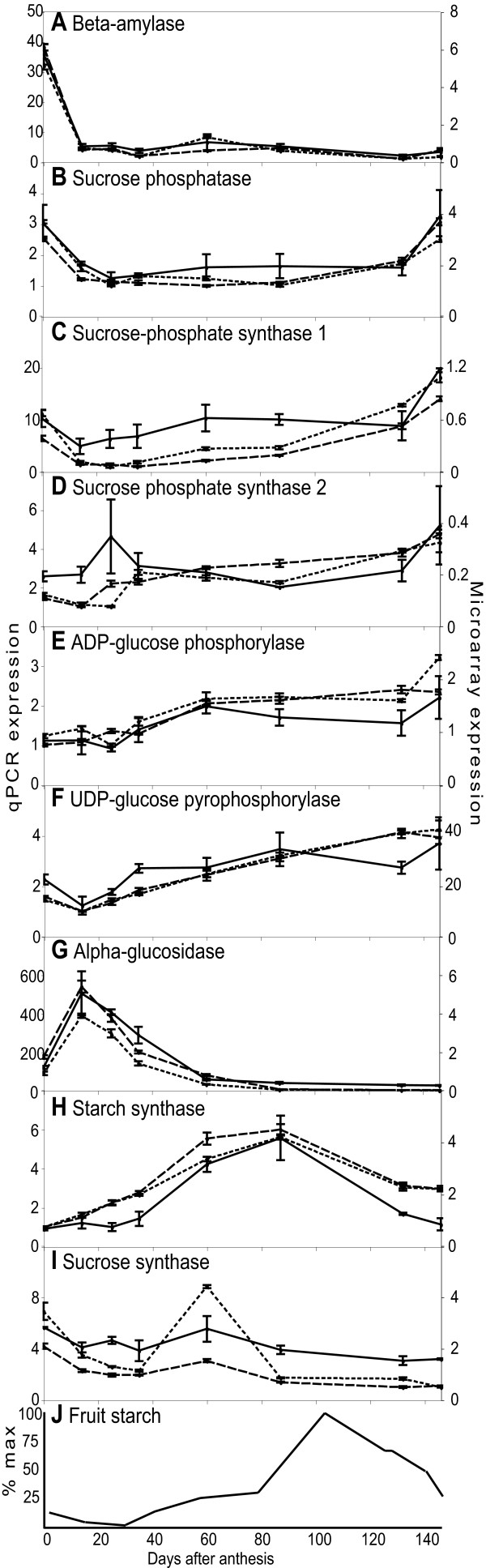
**Expression of starch metabolism genes**. Starch metabolic enzymes identified from KEGG were used to identify apple homologues. Where apple array expression varied and gave reliable data the expression pattern was confirmed by qRT-PCR. Of the 15 genes validated, 9 showed very similar patterns of expression in both array and qRT-PCR. A to F, The array data for Rep1 and Rep2 was combined and mean and standard error is plotted (solid lines), qRT-PCR data is shown for each Rep as mean and standard error for qRT-PCR replicates, Rep1 short dashes, Rep2 long dashes. G, Diagram showing fruit starch levels during fruit development as a percentage of the maximum levels, adapted from Brookfield et al. [19]. X axes show DAA, the left Y axes shows relative qRT-PCR expression; the right Y axes shows absolute array expression.

Genes which had constant expression during apple fruit development, and hence did not show transcriptional regulation in this developmental process were not studied further. Those with low-level expression were also excluded due to the high variability observed where the targets have low signal intensity on the microarray. α-amylase is one example of an enzyme for which the transcript level detected was below the cut off value and consequently was not analysed further. In total, ESTs for 15 apple genes with homology to starch metabolic enzymes were identified with microarray expression profiles that varied during fruit development (Table 4) and qRT-PCR was performed to confirm these profiles. For nine of the 15 enzymes, the qRT-PCR analysis produced expression profiles that strongly supported the patterns seen in the microarray data (Figure 5). For the remaining six enzymes the qRT-PCR pattern differed from the microarray pattern possibly because the RT-PCR primers were amplifying different alleles or genes than those detected by the microarray oligo.

Four distinct expression profiles were observed: I) for a β-amylase gene (EB114557), transcript levels were high at anthesis and low for the rest of fruit development, sucrose synthase (CN897963) had a similar pattern of expression although with a less rapid decline in expression; II) for sucrose phosphatase (EB156512) and a sucrose-phosphate synthase gene (EB123469), transcript levels peaked at the earliest and latest time points; III) for ADP-glucose phosphorylase (CN884033) and UDP-glucose pyrophosphorylase (EG631379), transcript levels were lowest in the bud and increased during fruit development to reach a maximum in tree ripe apple; IV) for an α-glucosidase (EE663791) and a starch synthase (EB121923) transcript levels were low both early and late in apple development and peaked during early and mid development, respectively.

Microarray data can potentially be used to identify regulatory genes associated with coordinating expression of pathways such as starch metabolism. The similarity of the profiles for sucrose phosphatase and sucrose-phosphate synthase (Figure 5) suggested coordination of expression. Using cluster analysis, a single domain Myb transcription factor (EB129522) was identified with a similar expression pattern to sucrose phosphatase and sucrose-phosphate synthase. Preliminary transient expression studies in *Nicotiana benthamiana *leaves did not show activation of promoter regions of the two starch metabolic genes using this Myb gene alone (data not shown). Further analysis using larger promoter regions and possible binding partners for the Myb protein may identify a regulatory role for this gene.

### Expression of candidate fruit development genes in apple

**Figure 6:**
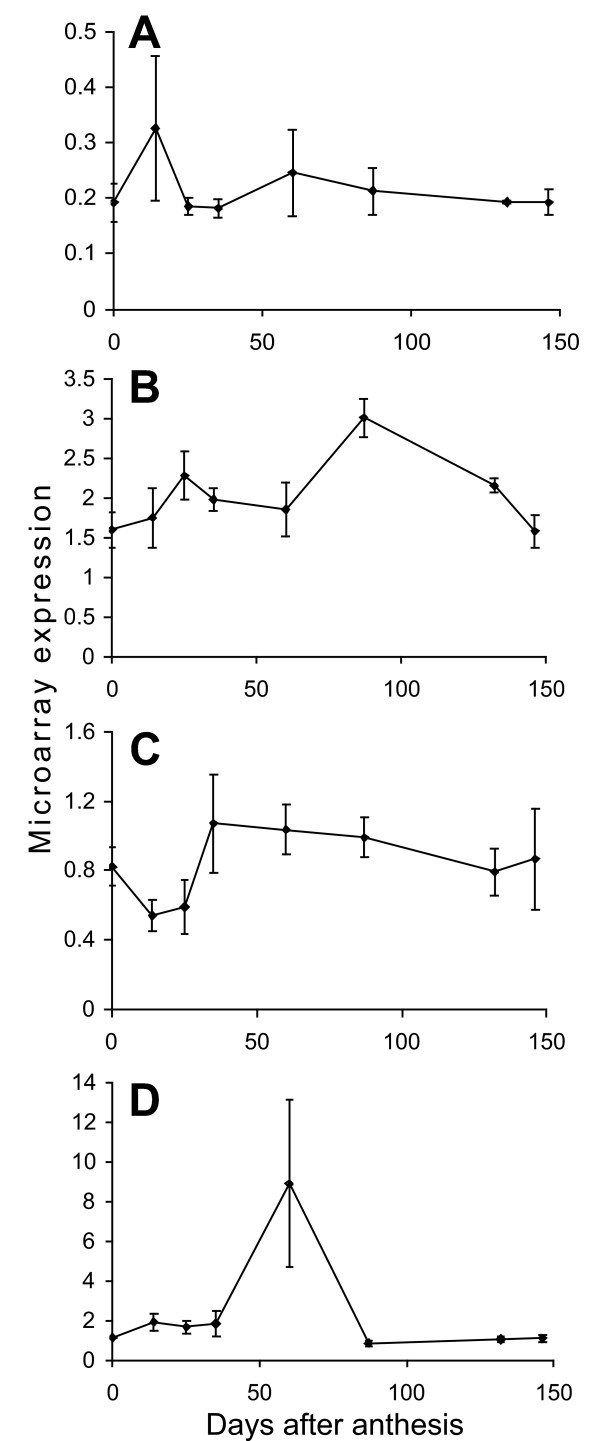
**Expression pattern for candidate fruit development genes**. Array expression patterns for apple homologues of Arabidopsis fruit development genes A, Spatula homologue EB132541, B, ettin/ARF3 homologue CN911459, C, Fruitfull/AGL8 homologue EE663894, D, Yabby homologue EB124712.

While Arabidopsis does not produce a large fleshy fruit and the post-pollination development of the fruiting body is limited, the availability of excellent genetic resources and genomic tools such as a complete genome sequence and whole genome microarrays has allowed identification of many important genes involved in floral and fruit development. The development of floral organs and the genes involved in production of mature carpels prior to fertilization have been the subject of several reviews [33]. Post-pollination development of the Arabidopsis fruit is limited, and while it serves as a good model for dehiscent fruit, it is not clear whether the genes involved in Arabidopsis fruit development are important in the development of fleshy fruit. In spite of this reservation, the importance of transcription factors such as *agamous*, *fruitful*, *AGL1/AGL5*, *spatula*, *crabs claw*, and *ettin *in specification of carpel identity and silique development suggests that transcription factors such as these may play significant roles in the development of fleshy fruit [33]. BLAST searches identified apple genes that had oligos on the apple microarray for a *spatula *homologue (At4g36930, apple EST289091 Genbank acc EB132541, expect value 8 × e^-41^); *ettin/ARF3 *(At2g33860, apple EST250932, Genbank acc CN911459, expect value 1 × e^-163^); a *fruitful/AGL8 *homologue (At5g60910, apple EST158712, Genbank acc EE663894, expect value 7 × e^-60^) and a *crabs claw *homologue (most homologous to *yabby5 *At2g26580, apple EST111296, Genbank acc EB124712, expect value 3 × e^-42^) and expression patterns for these genes were plotted (Figure 6). The expression of the *fruitful*/*AGL8 *homologue (Figure 6C), which has more similarity to AP1 than *fruitful*, increases at the time when apple fruit are enlarging (and down-regulated during cell division) which is interesting given the short compact silique of the *fruitful *mutant.

### Comparison of apple and tomato fruit development

A recent study by Alba et al. [13] used an array of 12899 EST clones representing ~8500 tomato genes to examine fruit development and ripening, with a particular focus on the events occurring around ripening. While this study did not include floral buds or the stages of tomato development, where cell division is most active, it is the most complete fruit development data set to date. In order to identify genes involved in both apple and tomato fruit development, we used the list of genes that change during tomato fruit development to find apple genes on our microarray.

Using MegaBLAST (word size 12, threshold 1 × e^-5^) the list of 869 genes that change during tomato fruit development from Alba et al. [13] was used to identify homologous apple genes that were present on the array used in this work. Three hundred and thirty-six unique tomato genes had homology to 479 unique apple genes by these criteria. Of these apple genes, 102 were identified as having significant changes in expression during apple fruit development and hence are transcriptionally regulated in both apple and tomato. We further filtered the list to include only those genes in the apple EFD (41 genes), MD (16 genes) and R (35 genes) clusters (Table 5). An additional 10 apple genes in the FB cluster were also identified by homology with the developmentally regulated tomato genes but not examined further since the tomato microarray did not include a floral bud sample.

**Table 5:** Comparison of tomato and apple fruit development genes

SGN-U ID (build 200607)^a^	TOM1 SGN-M ID^b^	Apple Genbank acc.	Putative Annotation^c^	e value^d^
**Early fruit development cluster**				

SGN-U313081	1-1-1.4.4.1	CN949202	Tubulin	4.00E-114
SGN-U334957	1-1-1.4.2.16	EG631180	dimethyllallyl pyrophosphate isomerase	2.00E-70
SGN-U313439	1-1-1.2.10.21	CN929316	Catalase isozyme	5.00E-67
SGN-U312411	1-1-3.1.20.8	CN929316	Catalase isozyme	1.00E-39
SGN-U314745	1-1-6.2.2.12	EB129157	Histone H2B family	5.00E-64
SGN-U315396	1-1-1.1.2.14	CN897140	Histone H2B family	1.00E-52
SGN-U320099	1-1-2.2.8.13	EB134184	homeodomain leucine zipper protein	5.00E-50
SGN-U312336	1-1-3.2.14.10	CN900880	Chlorophyll a/b binding protein CP24	8.00E-45
SGN-U316933	1-1-2.2.10.18	CN938965	SLT1 protein	1.00E-42
SGN-U312305	1-1-4.1.9.2	EB115858	Tubulin	5.00E-42
SGN-U312306	1-1-1.1.17.12	CN898685	Tubulin	3.00E-37
SGN-U312504	1-1-4.2.1.21	CN929029	Glycolate oxidase	5.00E-33
SGN-U312724	1-1-3.2.1.14	CN929029	Glycolate oxidase	9.00E-22
SGN-U313531	1-1-5.3.20.16	EB140736	multi-copper oxidase type I family protein	5.00E-33
SGN-U314489	1-1-5.4.1.13	EB128513	β-glucosidase	5.00E-30
SGN-U313179	1-1-3.3.12.5	EB149714	Photosystem I reaction center subunit N	3.00E-29
SGN-U313648	1-1-1.1.2.9	EB139544	multi-copper oxidase type I family protein	2.00E-26
SGN-U314548	1-1-1.1.14.13	EB128647	Peptidyl-prolyl cis-trans isomerase A	6.00E-25
SGN-U312538	1-1-1.3.12.16	EB130656	60 kDa chaperonin 2 (groEL protein 1)	8.00E-23
SGN-U312683	1-1-2.1.6.18	CN900931	Calreticulin precursor	9.00E-19
SGN-U319738	1-1-1.2.11.21	CN865336	zinc (C3HC4-type RING finger) family	3.00E-18
SGN-U314473	1-1-8.2.16.2	EB176490	MADS-box protein (AGL3) RIN	3.00E-17
SGN-U317999	1-1-4.3.10.21	CN945062	PGR5 related	8.00E-17
SGN-U318625	1-1-2.3.5.9	EB114733	kinase-activating protein	3.00E-16
SGN-U312874	1-1-1.3.11.19	CN909851	HMG protein	7.00E-16
SGN-U313470	1-1-2.1.19.16	CN940020	Hypothetical protein	2.00E-13
SGN-U333609	1-1-3.1.10.16	EB140812	expansin (EXP15)	7.00E-12
SGN-U313166	1-1-6.1.9.20	EB131083	Hypothetical protein	2.00E-11
SGN-U314384	1-1-5.4.4.11	EB132156	Lipid transfer protein (LTP1)	1.00E-10
SGN-U314386	1-1-5.1.15.12	EB132156	Lipid transfer protein (LTP1)	3.00E-07
SGN-U313194	1-1-2.3.4.21	EB131105	Photosystem I reaction center subunit psaK	3.00E-10
SGN-U313424	1-1-1.3.1.15	CN948056	seed storage/lipid transfer protein family	4.00E-10
SGN-U314489	1-1-5.4.1.13	EB141224	β-glucosidase, protein	1.00E-09
SGN-U312690	1-1-2.1.2.8	EB141004	Plastocyanin	2.00E-09
SGN-U336943	1-1-8.2.6.16	CN911937	hypothetical protein	7.00E-09
SGN-U331028	1-1-5.3.5.7	CN913037	Hypothetical protein	2.00E-08
SGN-U317844	1-1-8.4.6.17	EB140002	subtilase family protein	3.00E-07
SGN-U312690	1-1-2.1.2.8	EB127862	Glycolate oxidase^e^	4.00E-07
SGN-U313570	1-1-1.1.12.3	CN909757	hypothetical protein^f^	4.00E-07
SGN-U316057	1-1-6.4.13.2	CN882413	Aspartyl protease family protein	8.00E-07
SGN-U334601	1-1-8.4.10.14	CN887130	Aldehyde dehydrogenase 2B4	2.00E-06
SGN-U319033	1-1-3.2.20.7	EB133081	bZIP transcription factor	2.00E-06
SGN-U314713	1-1-1.2.1.20	CN918915	aldo/keto reductase family^g^	2.00E-06
SGN-U314261	1-1-7.4.10.14	EB148186	photosystem I subunit III precursor	6.00E-06

**Mid development cluster**				

SGN-U312527	1-1-4.2.20.9	EB130137	S-adenosylmethionine synthetase	8.00E-109
SGN-U312579	1-1-4.4.6.16	EB130137	S-adenosylmethionine synthetase	4.00E-70
SGN-U313529	1-1-6.3.1.18	EB130137	S-adenosylmethionine synthetase	6.00E-75
SGN-U313179	1-1-3.3.12.5	EB148119	Photosystem I reaction centre subunit N	4.00E-47
SGN-U312700	1-1-2.4.10.20	EB110724	Aquaporin PIP1.1	9.00E-46
SGN-U313179	1-1-3.3.12.5	EB138262	Photosystem I reaction center subunit)	2.00E-42
SGN-U313283	1-1-2.1.14.13	EB109090	Peptidyl-prolyl cis-trans isomerase	1.00E-37
SGN-U312814	1-1-3.3.9.20	CN943669	Plasma membrane intrinsic protein	5.00E-35
SGN-U316986	1-1-3.1.2.11	EG631337	class II heat shock protein	6.00E-33
SGN-U313962	1-1-5.2.4.10	EB143575	Hypersensitive induced response protein	7.00E-28
SGN-U312403	1-1-2.2.19.9	EE663740	Heat shock 70 kDa protein	1.00E-18
SGN-U313542	1-1-3.4.1.6	CN882970	plasma membrane protein	8.00E-18
SGN-U312953	1-1-3.3.3.13	EB129432	α-expansin precursor	4.00E-17
SGN-U333609	1-1-3.1.10.16	EB129432	α-expansin precursor	2.00E-06
SGN-U314790	1-1-6.3.18.20	CN913939	quinone-oxidoreductase protein	4.00E-17
SGN-U314793	1-1-2.3.17.10	CN913939	quinone-oxidoreductase protein	2.00E-10
SGN-U312450	1-1-7.3.19.9	EE663684	17.6 kDa class I heat shock protein	2.00E-12
SGN-U315846	1-1-3.2.11.11	CN866618	CBL-interacting protein kinase	2.00E-11
SGN-U314303	1-1-4.4.8.10	EB138124	Fatty aldehyde dehydrogenase	2.00E-10
SGN-U318440	1-1-8.1.15.21	CN875978	Hypothetical protein	4.00E-08

**Ripening cluster**				

SGN-U312527	1-1-4.2.20.9	EB137890	S-adenosylmethionine synthetase 1	6.00E-88
SGN-U312579	1-1-4.4.6.16	EB137890	S-adenosylmethionine synthetase 1	5.00E-42
SGN-U313529	1-1-6.3.1.18	EB137890	S-adenosylmethionine synthetase 1	3.00E-86
SGN-U312306	1-1-1.1.17.12	CN943168	Tubulin	5.00E-54
SGN-U314314	1-1-5.2.14.12	CN907169	Hypothetical protein	4.00E-44
SGN-U315828	1-1-3.2.1.16	CN940740	Cytochrome C oxidase subunit protein	5.00E-41
SGN-U334905	1-1-4.1.6.7	EB130234	β-carotene hydroxylase	2.00E-39
SGN-U312904	1-1-1.3.13.18	EB150480	haloacid dehalogenase hydrolase family	6.00E-38
SGN-U314358	1-1-4.3.1.2	CN915191	Alcohol dehydrogenase	5.00E-33
SGN-U319942	1-1-4.4.2.20	CN874208	Membrane-anchored ubiquitin-fold protein	2.00E-24
SGN-U316057	1-1-6.4.13.2	CN879999	aspartyl protease family protein	3.00E-22
SGN-U317374	1-1-8.2.2.7	CN946592	Hypothetical protein	3.00E-19
SGN-U336133	1-1-1.4.10.1	EG631183	α-amylase	5.00E-19
SGN-U318901	1-1-1.3.6.2	CN876487	Hypothetical protein	2.00E-17
SGN-U316698	1-1-3.2.1.19	CN868148	Seed maturation protein	5.00E-17
SGN-U316057	1-1-6.4.13.2	CN894718	aspartyl protease family protein	9.00E-16
SGN-U313923	1-1-4.2.19.5	CN883582	SNF1 protein kinase regulatory gamma	9.00E-16
SGN-U314101	1-1-2.4.13.5	CN941714	Chaperone clpB	7.00E-15
SGN-U317462	1-1-2.4.16.8	CN884487	Dual specificity protein phosphatase 6	5.00E-13
SGN-U313514	1-1-4.2.3.20	EB152301	14-3-3 protein GF14 upsilon (GRF5)	2.00E-12
SGN-U313747	1-1-2.3.3.5	EB128426	vacuolar processing enzyme-1b	3.00E-12
SGN-U316038	1-1-3.1.9.11	EE663883	Expressed protein	9.00E-12
SGN-U314449	1-1-8.1.4.18	CN902741	hypothetical or unknown protein	2.00E-11
SGN-U314453	1-1-2.4.16.1	CN902741	hypothetical or unknown protein	4.00E-11
SGN-U313315	1-1-3.1.9.21	EG631213	Putative chloroplast-targeted β-amylase	1.00E-09
SGN-U328474	1-1-8.4.1.16	CN911230	NHL repeat-containing protein	2.00E-09
SGN-U314887	1-1-3.3.3.14	EB144737	Phytoene synthase	3.00E-09
SGN-U313474	1-1-3.1.12.20	CN898201	short chain dehydrogenase/reductase family	3.00E-08
SGN-U322411	1-1-6.1.18.17	EB137522	Homocysteine S methyltransferase 1	2.00E-07
SGN-U315858	1-1-5.3.11.3	CN895375	Universal stress protein	2.00E-07
SGN-U315671	1-1-1.2.16.10	CN929435	Ethylene-responsive DEAD box RNA helicase	3.00E-07
SGN-U312714	1-1-2.3.9.4	EG631274	Cytochrome P450 85A1 (C6-oxidase)	2.00E-07
SGN-U312715	1-1-1.1.15.15	EG631274	Cytochrome P450 85A1 (C6-oxidase)	3.00E-07
SGN-U313547	1-1-2.4.5.5	CN917878	Plasma membrane ATPase 1 (Proton pump 1)	4.00E-07
SGN-U312870	1-1-4.2.15.8	EE663893	Xyloglucan:xyloglucosyl transferase	6.00E-07
SGN-U316695	1-1-1.2.8.9	EB111007	Mitogen-activated protein kinase 3	7.00E-07
SGN-U320099	1-1-2.2.8.13	EB116421	Homeobox leucine zipper protein ATHB-4	2.00E-06
SGN-U312516	1-1-1.3.7.19	EG631323	N-benzoyltransferase protein	4.00E-06
SGN-U312884	1-1-8.3.6.6	CN862135	Hypothetical protein	6.00E-06

Genes identified as changing during tomato fruit development were used to identify apple genes present on the array that were also changing during fruit development.

a Gene identifier for the tomato gene containing the sequence on the TOM1 array, from [53]

b Micrarray feature identifier from Alba et al. [13].

c Annotation of both the apple and tomato genes, based on BLAST comparison of genes with public databases.

d e value for the MegaBLAST comparison between the tomato gene and the apple gene that contain the sequence on the array.

e Annotation for tomato gene is: Plastocyanin, chloroplast precursor.

f Annotation for tomato gene is: Histone H4.

g Annotation for tomato gene is: protein transporter.

The expression data from both the apple and tomato microarrays was plotted for several of the genes identified. The top five genes in each cluster by quality of the BLAST match between apple and tomato were plotted. Several genes possibly involved in processes occurring during early fruit development, mid development and ripening were also plotted. And because microarrays have the potential to identify genes involved in processes without prior information, all the genes without annotation were also plotted.

**Figure 7:**
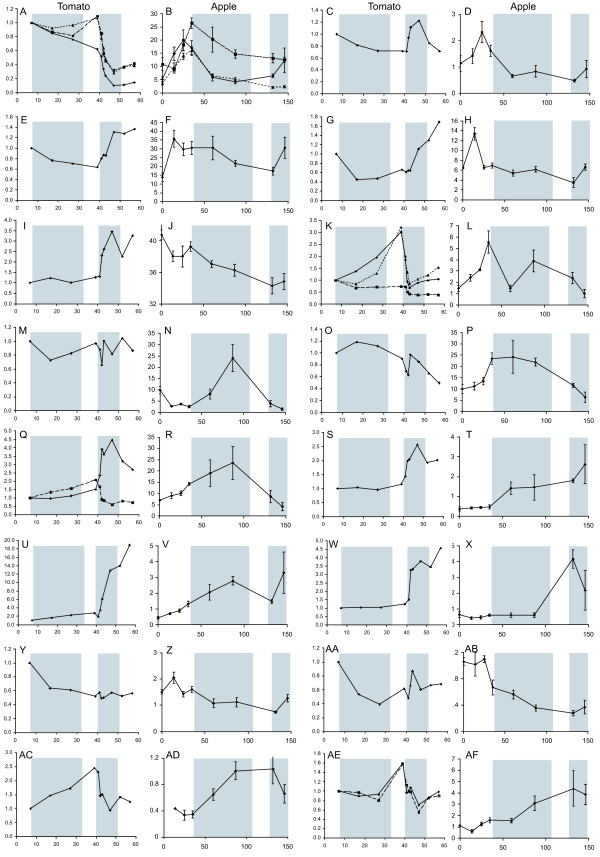
**Comparison of apple and tomato expression**. Expression of tomato and apple genes identified as changing during fruit development and similar by sequence comparison. Expression for tomato genes is plotted relative to 7 DAA and for apple as absolute expression; the x axes shows days after anthesis. Shaded areas in each graph correspond to the periods of cell expansion and ripening for both tomato and apple. A, C, E, G, I, K, M, O, Q, S, U, W, Y, AA, AC, AE tomato genes B, D, F, H, J, L, N, P, R, T, V, X, Z, AB, AD, AF apple genes. A and B, Tubulin homologues; C and D, IPP isomerase homologues; E and F, Catalase homologues; G and H, Histone 2B homologues; I and J, MADS box (RIN) homologues; K and L, SAM synthase homologues; M and N, PPIase homologues; O and P, plasma membrane protein; Q, and R, α-expansin homologues; S and T, β-carotene hydroxylase homologues; U and V, Alcohol dehydrogenase homologues; W and X Phytoene synthase homologues; Y to AF Unannotated proteins. A, solid line SGN-U313081, dashed line SGN-U312305, dotted line SGN-U312306; B, solid line CN949202, dashed line EB115858, dotted line CN898685; C, SGN-U334957; D, EG631180; E, SGN-U313439; F, CN929316; G, SGN-U315396; H, CN897140; I SGN-U314473; J, EB176490; K, solid line SGN-U312527, dashed line SGN-U312579, dotted line SGN-U313529; L, EB130137; M, SGN-U313283; N, EB109090; O, SGN-U312814; P, CN943669; Q, solid line SGN-U312953, dashed line SGN-U333609; R, EB129432; S, SGN-U334905; T, EB130234; U, SGN-U314358; V, CN915191; W, SGN-U314887; X, EB144737; Y, SGN-U317999; Z, CN945062; AA, SGN-U313570; AB, CN909757; AC, SGN-U318901; AD, CN876487; AE, solid line SGN-U314449, dashed line SGN-U314453; AF, CN902741.

The development of apple and tomato fruit, from anthesis to mature fruit differs in length, however we compared patterns of expression during similar phases of development, in particular the mid development phase when cells are expanding in both apple and tomato (~8–35 DAA in tomato and ~40–110 DAA in apple) and the ripening phase (~40–50 DAA in tomato and ~130–150 DAA in apple). Of the 47 genes for which expression patterns were compared, 16 had similar patterns of expression in both apple and tomato and are shown in Figure 7, with the cell expansion and ripening stages highlighted. A further five genes had some similarity of expression but 26 had little or no similarity of expression (data not shown).

For genes such as Tubulin (Figure 7A and 7B), SAM synthase (Figure 7K and 7L) and an expansin homologue (Figure 7Q and 7R) more than one tomato sequence had homology to an apple gene and in the case of the tubulin genes to three apple genes. For the tubulin genes the patterns of expression mostly differed between apple and tomato but one of the tomato genes showed a steady decrease in expression during cell expansion similar to the apple genes. For the three tomato SAM synthase genes only one (SGN-U312579) had a pattern of expression similar to the apple gene suggesting this tomato gene may have a similar function in apple and tomato. For the two tomato expansin homologues with similarity to apple, SGN-U312953 increased in expression during ripening whereas SGN-U333609 and the apple expansin homologue both increased during cell expansion and declined in ripening, suggesting these genes may be orthologues and have a role during cell enlargement but not in fruit softening. Four genes without annotation were identified as having similar patterns of expression in apple and tomato fruit. Further bioinformatic analysis suggests that, CN945062 may be a PGR5 homologue involved in photosynthesis, CN909757 is likely to be an F-box protein, and CN876487 which is expressed during cell expansion is similar to Sec5A and may be involved in exocytosis. However CN902741 still remains unannotated. The role of these genes in fruit development remains to be determined.

### Comparison of gene expression between apple cultivars

A recent report has examined expression of apple genes early in fruit development using an array of 3484 cDNAs [25]. These authors identified 88 unique apple genes expressed more in whole young fruit (21 DAA) than in whole mature fruit (175 DAA) in the cultivar Fuji. Eighty-four homologues of these genes were identified in our EST database, 42 of these were represented on our microarray. Of these 42 genes, 17 were selected as changing significantly during fruit development, 13 in the EFD cluster and four in the MD cluster (Table 6).

**Table 6:** Early apple fruit gene identified in 'Fuji' which change during 'Royal Gala' fruit development

EFD genes from Lee et al. (2007)^a^	expect value^b^	Genbank acc for array oligo	Annotation
**EFD cluster**			

DW248931	1.00E-155	CN900880	chlorophyll A-B binding protein (LHCI type I (CAB))
DW248987	0	EB127862	Glycolate oxidase
DW248917	1.00E-177	EB127279	lipid protein
DW248920	0	EB148186	Photosystem I reaction center subunit III
DW248842	0	CN929029	Glycolate oxidase
DW248924	0	EB115972	Ascorbate peroxidase
DW248835	0	EB140491	aquaporin TIP1.3
DW248922	0	EB149714	Photosystem I reaction center subunit N
DW248839	0	CN926591	NADH dehydrogenase
DW248976	0	EB112578	Trans-cinnamate 4-monooxygenase (Cytochrome P450 73)
DW248868	0	CN915536	rapid alkalinization factor
DW248881	1.00E-143	CN861574	phytol kinase 2
DW248942	0	CN861788	Photosystem I reaction center subunit V

**MD cluster**			

DW248803	1.00E-87	CN913162	CP12 protein
DW248895	5.00E-95	EB148680	Oxygen-evolving enhancer protein
DW248912	5.00E-163	CN870279	16.9 kDa class I heat shock protein
DW248912	5.00E-163	EG631337	class I heat shock protein

**Not selected in Royal Gala fruit development**			

DW248927	0	EB127218	Polyphenol oxidase
DW248967	0	EB127720	Ferredoxin-thioredoxin reductase
DW248839	0	CN894409	NADH dehydrogenase
DW248924	0	EB138975	Ascorbate peroxidase
DW248940	0	CN899704	oligouridylate binding protein
DW248833	0	EB128528	Hypothetical protein
DW248979	0	EB129884	α-expansin
DW248918	0	CN944949	Photosystem I reaction center subunit II
DW248941	0	CN884411	chlorophyll A-B binding protein
DW248844	0	EB148603	RuBisCO activase
DW248914	0	EB148603	RuBisCO activase
DW248854	1.00E-129	EB148750	Oxygen-evolving enhancer protein 2
DW248983	0	EB131218	fatty acid elongase 3-ketoacyl-CoA synthase 1
DW248994	0	CN912337	Glutamate-1-semialdehyde 2,1-aminomutase

Genes identified by [25] as up-regulated during EFD were used to identify apple genes present on the array.

a Genbank accession for those genes identified in Lee at al as up regulated in early fruit development with homologues present on our array.

b expect value for the BLAST comparison between the Fuji gene and the apple gene which contains the array oligo.

Since the criteria used to select significantly changing genes was fairly stringent we plotted expression patterns for all the matches between our data and the selected early fruit development genes from Lee et al. [25] in order to identify any additional genes with similar patterns of expression (data not shown). Eighteen genes identified by Lee et al. [25] as being up-regulated were not confirmed in our microarray, however, an additional 13 genes were identified with high expression early in Royal Gala fruit development, and low expression in ripening (Table 6).

### Identification of ethylene responsive fruit development genes

The hormone ethylene plays a major role in fruit ripening in many fruit, including apple, leading to the respiratory burst and final fruit softening [20,21,34,35]. Recent work has used a transgenic apple tree (expressing an antisense copy of the ACC oxidase gene) which produces no detectable ethylene to examine gene expression changes and production of volatile compounds associated with apple aroma [22]. Fruit from this tree mature, but do not ripen or soften, unless treated with exogenous ethylene. Schaffer et al. [22] used the apple oligonucleotide array described here to identify 944 apple cortex and skin genes that respond to ethylene. Because the ripe fruit samples in the fruit development experiment consisted of cortex tissue only we identified only those genes that change by at least 2-fold in cortex (after excluding 25 genes with very low expression), giving a list of 456 genes that respond strongly to ethylene in fruit cortex. Of these 456 ethylene-responsive genes, 106 also changed significantly during the ripening phase of normal fruit development. These ethylene-responsive fruit-cortex ripening genes are shown in Table 7 and are grouped by ripening sub-cluster. The distribution of the genes was uneven between the three clusters with a greater percentage of the R2 cluster also identified as ethylene responsive (10 of 70 genes (14.3%) in R1, 48 of 195 genes (24.6%) in R2 and 48 of 408 genes (11.8%) in R3). Included amongst these genes was one gene identified as a putative ethylene receptor, most similar to the ETR2/EIN4 receptors from Arabidopsis (apple EST166801, Genbank acc. EE663937). The apple microarray also contains oligonucleotide probes for four additional putative ethylene receptor genes. Expression of three of these genes was not significantly changed during the ethylene microarray experiment or during normal fruit ripening (apple EST152541, Genbank acc. CN898978, apple EST248756, Genbank acc. CN910963, apple EST244637, Genbank acc. CN902679). The fourth gene (apple EST166743, Genbank acc. EE663931, most similar to the ERS1/ETR1 receptors from Arabidopsis) was selected as induced by ethylene, and although it was not selected as significantly changing during fruit development, it does show some induction in normal fruit ripening.

**Table 7:** Fruit ripening genes which respond to ethylene

Apple Genbank acc.	Putative Annotation^a^	Apple Genbank acc.	Putative Annotation^a^
**Ripening sub-cluster R1**			

EB118159	Short chain dehydrogenase/reductase (SDR)	EB140551	Hypothetical protein
CN860849	Ceramide kinase	CN906574	Senescence associated protein
CN870499	Hypothetical protein	EB122632	Thaumatin protein
EB127428	LEA family protein	CN911315	DNA binding bromodomain protein
CN895403	Integral membrane family protein	EB151414	Major latex protein (MLP)

**Ripening sub-cluster R2**		**Ripening sub-cluster R3**	

EB106359	Hypothetical protein	CN932083	Chloroplast 50S ribosomal protein L22
CN862135	Hypothetical protein	CN860052	5-oxoprolinase
EB114937	β-glucosidase precursor	CN860296	Hypothetical protein
CN862240	Transaldolase ToTAL2	CN862389	Stress-responsive protein
EB116078	(S)-acetone-cyanohydrin lyase	CN864680	(S)-2-hydroxy-acid oxidase
EB118291	Mannitol dehydrogenase	CN851072	Calcineurin B-like protein
CN849429	Hypothetical protein	EB121320	Ribosomal protein
CN863631	Sugar transporter	CN886293	Isoflavone reductase
EB117418	(1–4)-β-mannan endohydrolase	EB135086	Carbonic anhydrase
CN864737	DNA polymerase III polC-type	EB126988	C2H2-type zinc finger protein
EB115757	Flavonol synthase	CN939170	Glycerol-3-phosphate dehydrogenase
EB121772	Hypothetical protein	CN939718	Sad1/unc-84 protein
CN887217	Hypothetical protein	CN890306	Transaldolase protein
CN890755	CBL-interacting protein kinase	EB137446	Cytochrome P450
EB135512	F-box family protein	CN894690	NADH dehydrogenase
CN893578	C-4 methyl sterol oxidase	EB137890	S-adenosylmethionine synthetase
CN889902	Auxin/aluminum-responsive	CN895673	2-oxoisovalerate dehydrogenase
CN895410	Hypothetical protein	EB138408	Hypothetical protein
CN895502	Vacuolar sorting receptor	EB140312	Ribose-5-phosphate isomerase A
EB138209	Xyloglucan endotransglycosylase	EB142251	Pectinacetylesterase
EB138429	LEA family protein	CN941807	DEAD box RNA helicase
CN940062	Harpin induced protein (HIN1)	CN943134	Hypothetical protein
EB139752	Seed storage/lipid transfer protein	EB129495	Stress-responsive protein
EB139896	Auxin-responsive protein	CN943168	Tubulin
EB128540	Profilin	EB129522	MYB transcription factor
CN943110	Syntaxin	CN945056	Hypothetical protein
EE663937	Ethylene receptor (EIN4/ETR2)	CN883038	Hypothetical protein
EB140933	6-phosphogluconolactonase	EB150480	Haloacid dehalogenase hydrolase
EB141282	Lipoxygenase	EE663647	Hypothetical protein
CN901620	Hypothetical protein	EG631194	S-adenosyl-L-methionine:carboxyl methyltransferase protein
EB144781	Lipid transfer protein	CN876100	SCARECROW gene regulator
EB148006	Fimbrin protein (FIM1)	CN877052	Hypothetical protein
EG631181	Thaumatin protein	CN878203	Copine I protein
EG631195	Transferase family protein	CN902180	Amidase protein
EG631213	β-amylase	CN902277	Hypothetical protein
EB157538	Hypothetical protein	CN902592	Heavy-metal associated domain-containing protein
CN875931	tatD deoxyribonuclease family	CN911536	Hypothetical protein
EG631252	UDP-glucoronosyl/UDP-glucosyl transferase	EB154218	MADS-box protein
EE663809	Pyruvate kinase	CN914798	Hypothetical protein
CN912930	Pentatricopeptide repeat protein	CN914935	MATE efflux protein
CN913545	Hypothetical protein	CN914950	2OG-Fe(II) oxygenase family protein
CN909301	Hypothetical protein	CN917878	H(+)-transporting ATPase
CN917441	Dormancy/auxin associated	EE663837	Hypothetical protein
EB152801	Xyloglucan endotransglycosylase	CN916212	AAA-type ATPase family protein
CN915067	Sugar transporter family protein	CN916137	Phytase
CN915323	Hypothetical protein	EB153327	Isocitrate lyase
EE663891	Polygalacturonase	CN915191	Alcohol dehydrogenase
EG631317	Cytochrome P450	EG631278	Cytochrome P450

Apple genes for which expression changed in response to ethylene treatment of mature apple fruit from an ACC oxidase knockout plant [22] which also had significantly altered expression during fruit ripening in the fruit development array.

a Annotation of the apple genes, based on BLAST comparison of genes with public databases.

## Discussion

### Confirmation of microarray expression patterns by qRT-PCR

At each of the steps used to produce microarray data, variability can be introduced leading to potential errors. We used qRTPCR of cDNA from the same samples of RNA used in the microarray experiment itself to estimate the overall accuracy of our data. Overall we found good correlation between qRT-PCR and microarray results with 75% of microarray expression patterns reproducible by qRT-PCR. However, 25% of expression patterns for which the qRT-PCR results did not match the microarray result. In some cases (~5%), this difference seems to be associated with genes where the genomic DNA reference sample gave very high intensity binding. It is possible that this high level of gDNA binding distorted the ratios observed or the gDNA binding may have interfered with cDNA binding for those genes. Another possible explanation for qRT-PCR results disagreeing with microarray results is that the oligo on the microarray was able to hybridise to more than one allele of a gene in the sample, and qRT-PCR primer binding was more specific. Alternatively the microarray oligo may be hybridizing to more than one member of a gene family. These results would suggest that hybridization conditions on the microarray are not stringent enough, however during initial optimization of the methods any increase in stringency resulted in a large loss of signal intensity (data not shown). Furthermore, we have approximately 20 oligos on the array that were designed to EST sequences which when re-sequenced were shown to have a single base mismatch to the consensus sequence, these oligos do not bind labelled targets whereas perfect match oligos to the same targets do produce good signal (data not shown) suggesting hybridization stringencies are close to optimal.

### Different functional classes of genes are expressed at different times during fruit growth

A comparison between the whole array and the selected 1955 genes identified differences in distribution of functional categories, suggests that the genes selected as changing significantly is a non-random selection from the whole array. The increases in "metabolism" and "energy" classes as compared with the whole array are not unreasonable given the large changes occurring in organ development and the accumulation of starch and sugar and later in ripening and production of flavour compounds. The remaining functional classes show only minor differences between the whole array and the selected 1955 and this may reflect some bias in the EST sequences [24]. Since the majority of libraries used in the original EST sequencing were from fruit or floral buds, it is reasonable to expect functional classification of the whole array to be similar to the classification for those genes regulated in fruit development.

When the functional classifications for the four major clusters are compared, some interesting changes in the proportions of genes in each category are observed, although interpretation of these changes must be made with caution since each cluster represents a different set of genes. The proportion of metabolic and energy gene functions is high in buds and declines during development and then increases in ripening fruit. This late increase may reflect an increase in secondary metabolite gene expression as flavour compounds are produced during ripening. An indication of this can been seen when the functional categories are examined in more detail. While the overall "metabolism" classifications are similar for FB and ripening clusters (21.5% vs 20.9%) the MIPS category 01.06 for lipid, fatty-acid and isoprenoid metabolism, which include the known flavour components such as terpenes, shifts from 2.6% in FB to 4.3% in the ripening cluster (data not shown).

A limitation of functional analysis is that it can only provide information about genes for which some function has been previously identified. While functional classification of genes is a useful approach to analysis of microarrays it is the combination of functional classification with other approaches (e.g. clustering) that allows information to be more easily identified in the data, for example identifying genes associated with cell division that are most highly expressed early in development.

### Cell cycle genes are regulated at the transcriptional level early in fruit development

The development of apple fruit involves an early period of cell division that lasts for approximately 30 days after pollination [17,18]. Regulation of cell cycle genes is complex however it is possible that transcriptional regulation of some of the core cell cycle genes are involved in the control of cell division during fruit development. Control of the core plant cell cycle genes at the transcriptional level has been associated with regulation of the cell cycle in synchronised Arabidopsis and tobacco BY2 cell cultures [30,36-38]. Because of the nature of our samples, we would not be able to detect such cycle-dependent transcriptional regulation. However, at least one of the core cell cycle genes has been shown to be regulated developmentally in plants; CDKB1;1 has been associated with control of cell division in Arabidopsis leaf development, and expression of CDKB1;1 declines as Arabidopsis leaves get older [39,40]. Alteration of CDKB1;1 activity in leaves by expression of a modified form of CDKB1;1 changes cell size and endoreduplication. Two putative CDKB homologues in the apple fruit development microarray changed significantly, both of these apple genes decline in expression at the time that apple cell division stops suggesting a role for these genes in the regulation of this process. The third core cell cycle gene that changed significantly during fruit development is a CKS1 homologue. CKS1 has been shown to associate with CDKB proteins and has been proposed to act as a docking protein for regulators of CDK activity [41] and also has been shown to associate with the SCF complex involved in degradation of kinase inhibitor proteins (KIPs in animals, KRPs in plants, [41,42]). The expression of these three cell cycle associated genes at the time when apple fruit are undergoing cell division suggests they are important developmental regulators in apple. Altering expression of these genes would allow elucidation of their function and perhaps lead to fruit with altered cell numbers leading to changes in fruit texture and size.

The G1 to S transition is an important control point in the plant cell cycle and the CycD3;1 gene has been shown to be limiting for this transition in Arabidopsis [43]. No orthologue for this gene has been identified in apple although three homologous genes are represented on the array. None of these homologues varied significantly during development but one (EB132575) declined approximately 2-fold late in apple fruit development.

Endoreduplication has been associated with increases in cell size in many plants [44]. Studies in Arabidopsis suggest that inhibition of mitotic CDK complexes by the kinase inhibitors KRP1 [45] and KRP2 [46] and the kinase Wee1 [47] can lead to increased endoreduplication. Interestingly, a recent report suggests there is no endoreduplication in mature apple fruit [48]. Perhaps not surprisingly then, apple homologues of these genes were not selected as having changed significantly during fruit development, however the apple Wee1 homologue does show some increase in expression immediately after cell division ceases. The role of these genes in regulation of endoreduplication in apple, if any, is not clear but it may be possible to induce endoreduplication in apples by altering expression of these genes.

### Starch metabolism is regulated at the transcriptional level in fruit

Although the biochemical activities of many starch enzymes have been defined, it is difficult to assign the roles of different enzyme pathways in the regulation of starch levels in fruit. Matching the gene expression profiles produced in this study to known changes in starch content throughout apple development is one approach, implicating certain pathways in these processes. While we did not observe coordinated expression of complete pathways, there was co-expression of several genes in one pathway. For example, the expression profiles of sucrose phosphatase (EB156512) and a sucrose-phosphate synthase (EB123469) mirrored the reduction in apple starch content during both early fruit development and during ripening [19], suggesting that these enzymes may be components of the starch degradation pathway in fruit development. However, it is also possible that distinct pathways are responsible for these early and late starch degradation events. The high transcript levels of β-amylase (EB114557) and α-glucosidase (EE663791) early in development but not during ripening are evidence of a starch degradation pathway that may be specific to early development and not active in late development. These results suggest that distinct starch metabolic pathways are important and are regulated at the transcriptional level in apple fruit development.

One observation made during the analysis of the starch metabolism pathways was that for any given step there were usually several candidate genes for a particular enzyme. For example there are two plastidic starch synthases in the Arabidopsis databases. Both have homologues in the apple EST database, and one has homology to two apple genes. Expression of only one of these candidate starch synthase genes in apple fruit (represented by EB121923, Figure 5) peaked at 87 DAA, just prior to the peak in fruit starch content at 100 DAA [19]. This correlation of expression data with the pattern of starch accumulation during development suggests that this particular starch synthase gene is involved in regulation of starch levels during fruit development. These results show that microarrays can be used to correlate transcript levels with physiological and biochemical observations to identify which member of a gene family, or even perhaps which allele, is likely to be involved in the process of interest.

The expression profiles of nine starch enzymes (Figure 5) showed that developmental regulation of the transcription of these genes corresponds to observed changes in starch levels throughout apple development. In a similar study, Smith et al. [49] used Affymetrix microarrays to observe εchanges in the expression of starch enzymes over a diurnal cycle in Arabidopsis leaves. In leaves, starch is synthesised in the light and degraded in the dark. These authors observed distinct changes in the transcript levels of enzymes such as starch synthase and β-amylase. It is interesting that there is evidence of transcriptional regulation of starch in both Arabidopsis leaves where light- and sugar-regulated changes in starch occur over a 24-hr period, and in apple fruit where developmental regulation of starch takes place over a 146-day period. This transcriptional regulation of starch in both source and sink tissues may be required to coordinate the partitioning of carbohydrates throughout a plant.

### Comparison of microarray experiments examining fruit development

Comparison of microarray experiments from different species targeted to the same developmental process offers the opportunity to compare gene expression patterns for a large number of genes. The attraction of such a comparison is that it may identify processes common to different fruit and hence important in the fundamental processes occurring in all fruit. For some published studies however, the size of the datasets and/or differences in samples studied make comparisons of limited value [11,15,25]. For example, specific searches of the tomato microarray results given by Lemaire-Chamley et al. [11] for genes expressed in both apple (this work) and tomato [13] early in fruit development did not identify similar genes, probably because these genes were not included in the Lemaire-Chamley array of 1393 tomato cDNAs e.g. IPP isomerase homologues (SGN-U334957 and EG631180), catalase homologues (SGN-U313439 and CN929316) and Histone 2B homologues (SGN-U315396 and CN897140). Where the apple microarray identified a CDKB2 gene as up-regulated early in fruit development (dividing cells), a comparison of tomato locular (expanding cells) and tomato pericarp (dividing cells) identified a CDKB2;2 homologue as up-regulated in locular tissue [11].

A microarray experiment using apple (3484 cDNAs, 'Fuji') compared 21 DAA with fully ripe fruit [25]. Comparing our data with that of Lee et al. [25] allows identification of regulated genes that may be otherwise excluded as not significantly changing in one of the two experiments. One such gene is EB129884 an α-expansin homologue, identified as highly expressed in 21 DAA fruit in the Fuji microarray, was excluded from the Royal Gala microarray by ANOVA analysis because two samples (132 and 146 DAA) had no detectable expression. In the Royal Gala microarray this α-expansin had strongest expression at 14 DAA and maintains expression through to 87 DAA and then has no detectable expression, making it a good candidate for an expansin involved in the formation and expansion of the fruit cells. Without the comparative analysis with the data from the Fuji microarray this gene would not have been identified.

Using a microarray containing 12899 ESTs representing ~8500 tomato genes Alba et al. [13] studied gene expression through tomato fruit development, focusing predominantly on ripening. It was perhaps surprising to find only 102 genes in common between the tomato fruit development microarray and the apple data presented here. The differences in experimental design may be one reason for this small overlap, with the tomato microarray having more sampling around ripening and the apple microarray more sampling of the floral bud and early fruit development. It may also be an indicator of the differences between apple and tomato fruit development.

When expression patterns for the similar apple and tomato genes were compared, only 16 out of 46 genes studied had similar patterns of expression in both apple and tomato. Since approximately 75% of apple microarray expression patterns are reproducible in qRT-PCR, and presumably the same is true for the tomato microarray, for each pair of genes there is only an approximately 56% chance that both patterns are reproducible. Thus at best we would expect only 26 pairs to have the same pattern of expression. In addition, since the sequence similarity threshold used was fairly low it is also likely that some of the pairs of genes examined are not orthologous genes. Nevertheless it is likely that identifying only 16 pairs of genes with similar expression patterns in both apple and tomato is an underestimate of the actual similarity between the fruit. Where patterns of expression do have similarity between apple and tomato it is probable that the microarray pattern of expression represents the actual pattern of expression for those genes, since the expression pattern has effectively been confirmed in another species. It is probable that when more complete whole genome arrays are used and when more closely matched sampling is carried out, many more genes with similar expression will be identified. As further microarray experiments are performed in other fruiting species the inclusion of samples at standardized developmental stages will allow better comparison of datasets and more common fundamental processes to be identified.

Of the 16 pairs of tomato and apple genes identified, seven show up-regulation in ripening and four showed down-regulation. This almost certainly reflects the emphasis on ripening samples in the tomato microarray. Homologues of β-carotene hydroxylase, alcohol dehydrogenase and phytoene synthase are all up-regulated during ripening in both apple and tomato, suggesting these enzymes play significant roles in formation of the colour and flavour compounds associated with ripening fruit. However, carotenoids are not typically high in apple fruit flesh [50] suggesting either that production of carotenoids in apples is blocked at another step in the biosynthetic pathway or that the products of these enzymes are further processed into forms that have not yet been measured in apples. While homologues of IPP isomerase, catalase, Histone 2B and the RIN MADS-box gene are all up-regulated in ripening in both apple and tomato they were all also selected in the apple microarray as up-regulated early in fruit development, although for the MADS-box gene the up-regulation may be more associated with high expression in floral buds. The role of this early expression for these genes is uncertain but it would be interesting to see if they were also highly expressed early in tomato fruit development. One integral plasma membrane protein homologue and one expansin homologue showed similar patterns of expression in both apple and tomato and were selected in the mid development cluster in the apple microarray. This result suggests these two genes play important roles in cell expansion during fruit development. We also identified genes without annotation that have similar patterns of expression in both apple and tomato fruit. Such comparisons are valuable in order to find genes for which the function is conserved for a particular process that may not be identified by other methods. Further work will allow us to determine whether these genes indeed play an important role in fruit development.

### Intersections between different apple microarray experiments

A comparison between two apple experiments using the same microarray was useful to identify genes involved in both fruit ripening and the ethylene response. The combination of the two datasets provides more information than each experiment on its own. The importance of ethylene in apple fruit ripening is demonstrated by the lack of ripening in ACC oxidase knockout fruit [22]. When we compared datasets from the ethylene induction and the fruit development microarray, 106 of the ethylene induced genes (in cortex) were found in the ripening cluster (668 genes) of the developmental microarray. The observation that 350 of the ethylene induced genes were not identified as having altered expression during the endogenous ripening process implies that these genes do not have roles in normal fruit ripening, or that the induction of these genes is below the level of significance used to select genes in this work. These results suggest that while ethylene is a major regulator of gene expression in fruit ripening, a large portion of fruit ripening occurs in the absence of ethylene. Using this comparative approach it is possible to identify fruit ripening events that are both ethylene dependent and independent.

## Conclusion

The data presented here provide a picture of the molecular events occurring throughout the development of the apple fruit and provide a resource for future study of fruit development. We have identified genes that are likely to be important in some of the major processes. Comparison of the apple data with other fruiting plants identified 16 genes that may play fundamental roles in fruit development. Comparisons between experiments in apple allows differentiation between ethylene dependent and independent ripening. Future work will determine the specific function of these genes. Functional analysis of CDKB and CKS expression in fruit tissue early in development may reveal the mechanisms that control the growth of the cortex tissue to surround the core. Manipulation of expression of these genes may alter cell size and number in fruit, perhaps affecting fruit shape, size and texture. These data allow us to begin to develop an understanding of the molecular events that lead to the division and expansion of tissues surrounding a developing seed to form a fruit.

## Methods

### Growth and maintenance of trees and sampling

Apple (*Malus *× *domestica *Borkh. also known as *M. pumila*) trees from 'Royal Gala' were grown on M9 rootstocks and managed according to standard orchard practices (except that no chemical fruit thinning was allowed to take place).

For the 0 DAA sample, buds were stripped of petal and petiole but otherwise not further dissected. For samples taken at 14, 25, 35 and 60 DAA, whole fruit were sampled with only the petiole removed. For each sample at least 10 individual whole fruit were pooled. For samples taken at 87, 132 and 146 DAA cortex tissue only was dissected from at least 10 fruit and pooled. All samples were frozen in liquid nitrogen at time of harvest and then stored at -70 °C.

### RNA extraction

Total RNA was extracted from 6 g of each tissue ground under liquid N_2 _conditions using a modified method of Chang et al. [51]. The protocol was amended with a 1 min polytron step after addition of the extraction buffer, the aqueous phase after the first chloroform extraction was filtered through autoclaved Mira cloth, and the total concentration of LiCl was 2 M. Isolated RNA was column purified (using RNAeasy Mini Kit, Qiagen, Hilden, Germany) and the quality and purity was checked using an Agilent 2100 Bioanalyser (Agilent, Palo Alto, CA). RNA was ethanol precipitated and resuspended to 12.5 μg/μL.

### Array design

Apple ESTs were grouped into non-redundant sequences and unigenes as described in Newcomb et al. [24]. For each EST, oligonucleotides were designed using an in-house algorithm, with a Tm of 74°C ± 2°C and length between 45 and 55 bases. Oligos with inverted or direct repeats and runs of more than 5 identical nucleotides were eliminated. A single oligo was selected for each unigene from the EST closest to the 3' end of the unigene and where more than one possible oligo was available for an EST, the 3' most oligo selected. As a final selection criterion unigenes were compared (using BLAST) with the database of apple unigenes and to the Arabidopsis protein database. For apple unigenes with high sequence similarity to other apple unigenes or where two apple unigenes had high sequence similarity to the same Arabidopsis protein only a single representative apple unigene was selected for oligo design. Using these criteria 15726 apple oligos were designed corresponding to 15145 apple unigenes (Table 1). Comparison of the apple unigenes with Arabidopsis and other plants suggests that the array contains approximately 13000 different genes. Oligos were synthesized commercially (5000 by Operon and 10726 by Illumina). Oligos were resuspended in 150 mM NaPO4 pH 8.5 containing 0.00001% SDS to a final concentration of 20 μM and printed on epoxy array slides (Quantifoil) using a MicroGrid TAS arrayer using 16 microspot 2500 pins for a total of 32 blocks. Since oligos were selected and synthesized in random order, no additional randomization of the array was necessary.

In addition to the sample oligos, each block contains four types of control oligos (Table 8). Group 1, apple oligos designed from: the 3', middle and 5' ends of an apple actin unigene (MdAC1–3, EST3793, Genbank acc. CN935584), an apple ubiquitin unigene (MdAC4–6, EST14223, Genbank acc. EB109811) and an apple elongation factor-1-α gene (Md AC7–9, EST704, Genbank acc. CN934151); oligos from apple rubsico small subunit (MdAC10, EST 59854, CN862467); an apple homeobox unigene, 5' end (MdAC11, EST87558, Genbank acc. CN870331), 3' end (MdAC12 EST29626, Genbank acc. EB111272) and conserved domain (MdAC13, EST29626, Genbank acc. EB111272); an apple MADS-box gene, 3' untranslated region (MdAC14, EST58802, Genbank acc. EB175510), 3' coding region (MdAC15, EST64768, Genbank acc. EB116541) and MADS domain (MdAC16, EST15992, Genbank acc. EB114519).

**Table 8:** Control oligos

Control name	Apple EST	Genbank Acc.	TAIR acc.	
MdAC1	1412	EB106245		CGAACCAACACCAAAGGCCCTCAAGGCGGGCAGCATCACTACCAT
MdAC2	1412	EB106245		GCTCTTCCACATGCCATCTTGAGGCTTGACCTTGCAGGTCGTGAT
MdAC3	1412	EB106245		TACTTAAAATGTCTGGATTCTATGAGTTTGTAGGTTTGCCGCTGG
MdAC4	14223	EB109811		CTTCAATCTGAAAAATCTTCCTTCAAATTCTCTTTCCAAGCTTCTTCAGCC
MdAC5	14223	EB109811		TGAGGTGGAGAGCTCCGACACCATAGACAACGTGAAGGCCAAGATTCAAG
MdAC6	14223	EB109811		AATGGTACTGTTTTTGCCTCCTAAGATGAGGCATCTGGGCAAGTTTGTG
MdAC7	704	CN934151		CAACATCGTGGTCATTGGCCATGTCGACTCCGGCAAGTCGACCAC
MdAC8	704	CN934151		TGTTGAGACTGGTATCGTCAAGCCTGGTATGGTTGTGACTTTTGG
MdAC9	704	CN934151		GGTGGTGACCCATCAAGTTTATGTTGTGTCGATTCCGCCTTCTGA
MdAC10	59854	CN862467		GTGTTATGTATGCATAAGGAAGGTTATGGTTTATGCTGCTCCCTG
MdAC11	8626	CN923132		GCCATAAGCTTTAAGCTCTTCTCTCTGATTTCTCACAATTCAACTCGC
MdAC12	29626	EB111272		ACGAGCCTTGCACCAACCTTAATTTGAAAAGAAGTAATGCAAGTG
MdAC13	29626	EB111272		AAGACGATAAACAACTGGTTCATCAATCAGCGGAAGAGGAACTGG
MdAC14	58802	EB175510		CCTGGGTGGATGCTTTGACTTTGTTTGTGCCTAATAATAATACCC
MdAC15	64768	EB116541		GACTCTGGAACCATTATATGAATGCCATCTCGGATGCTTTGCTGC
MdAC16	15992	EB114519		ACGAATCGAGAACACGATAAGCAGGCAAGTGACATTCTCAAAGAG
BtAC17		U89872		TTCCAATTCACTTCCCATCGACATCTACCAGATATCGAGTTCGTG
ShAC18		X17220		CACCATCGTCAACCACTACATCGAGACAAGCACGGTCAACTTCCG
AvAC19		AF078810		GCCCTGTCCTTTTACCAGACAACCATTACCTGTCCACACAATCTG
HsAC20		NM_000518		GTGTGGCTAATGCCCTGGCCCACAAGTATCACTAAGCTCGCTTTC
EcAC21		A00196		TAACAAGAAAGGGATCTTCACTCGCGACCGCAAACCGAAGTCGGC
EcAC22		K01193		GTCTGGACCGATGGCTGTGTAGAAGTACTCGCCGATAGTGGAAAC
PpAC23		X65316		AGAGAGATCCTCATAAAGGCCAAGAAGGGCGGAAAGTCCAAATTG
EcAC24		V00618		TCGCAGCGCATCGCCTTCTATCGCCTTCTTGACGAGTTCTTCTGA
HsAC25		AF126021		CAGTGTTGTTCCCTCCCTCAAGGCTGGGAGGAGATAAACACCAAC
HsAC26		X13988		AAGAGTGAGCCAGCCCTTCTGGAGCAGGAGCAGGACAGAAGATAT
HsAC27		M21812		CACGCAGTGTGACCGCTTCTCCCAGGAGGAGATCAAGAACATGTG
HsAC28		X07868		TCAGCTCCTTTAACGCTAATATTTCCGGCAAAATCCCATGCTTGG
HsAC29		AK001779		GTGCCGGACTTACCTTTCATTGAACATGCTGCCATAACTTAGATT
HsAC30		AF161469		ATGCTTAAGATTCAACTGGGAGCATACCAGGGATGCTCTCTAACG
HsAC31		NM_004048		TGGCAACTTAGAGGTGGGGAGCAGAGAATTCTCTTATCCAACATC
HsAC32		NM_000291		GCTCATCTTCACTGCACCCTGGATTTGCATACATTCTTCAAGATC
HsAC33		L11329		GTGTCATGTTGCGTGTGTCTGTCTGTGAGCCTTTCACACCTGTGC
HsAC34		U11861		GAGTTGGAGCACGGTCTCTATGGGGAAGCGTTCGCTGTCTATCAG
Aunc1			At1g14400	GCTAACTCCTGATGGAGAGCTTTCGAAAATCAGTTGAATCAACCTCTGTT
Aunc2			At1g16210	GTCGATTTCATCATCATGTCCACCGATGTGCATTTGCAATTTGAAACGCAT
Aunc3			At1g43900	CCGGCTCAGAGTAAGGACTTGGATTCCTACCTTATTGGTAGGGTGGCGGTGC
Aunc4			At3g13060	GCCTGCCCGTGACGAGAGCGGTGCTACTATTAGGCATTTTACGAGTTAGCC
Aunc5			At3g19420	ATGCCTCCGTTTTCTCGTTTGGAGATGACGAGGACTCTGAAAGTGAGTAAACAAGG
Aunc6			At3g19760	CACAGAATTGGTCGTAGTGGACGTTTTGGAAGGAAGGGTGTTGCCATCAACTTCG
Aunc7			At4g00660	GCCGGTGATTGGTGGTGGAGAACCTTGATGTGACAGCAATGATGGGATGA

Group 2, control oligos associated with transgenic plants: *Bacillus thuringiensis *cry1Ac (BtAC17, Genbank acc. U89872); *Streptomyces hygroscopicus *phosphinothrycin acetyl transferase (ShAC18, Genbank acc. X17220); GFP (Av AC19, Genbank acc. AF078810); *Homo sapiens *hemoglobin (HsAC20, Genbank acc. NM_000518); GUS (EcAC21, Genbank acc. A00196); *E. coli *hygromycin B phosphotransferase (EcAC22, Genbank acc. K01193); luciferase (PpAC23, Genbank acc. X65316); Neomycin phosphotransferase (EcAC24, Genbank acc. V00618).

Group 3, control oligos from human genes not expected to be expressed in plants: B-cell receptor protein (HsAC25, Genbank acc. AF126021); Mysoin heavy chain (HsAC26, Genbank acc. X13988); Myosin reg. light chain 2 (HsAC27, Genbank acc. M21812); Insulin-like growth factor (HsAC28, Genbank acc. X07868); cDNA FLJ10917fis (HsAC29, Genbank acc. AK001779); HSPC120 (HsAC30, Genbank acc. AF161469); β2 microglobulin (HsAC31, Genbank acc. NM_004048); Phosphoglycerate kinase (HsAC32, Genbank acc. NM_000291); Tyrosine phosphatase (HsAC33, Genbank acc. L11329); G10 homolog edg-2 (HsAC34, Genbank acc. U11861).

Group 4, control oligos expected to have the same level of expression in all tissues based on analysis of Arabidopsis array data: Ubiquitin protein ligase (Aunc1, At1g14400, Genbank acc. T21817, ESTID103C16T7); unknown protein (Aunc2, At1g16210, Genbank acc. T04357, ESTID39A2T7); phosphatase (Aunc3, At1g43900, Genbank acc. H76500, ESTID196M23T7); unknown protein (Aunc4, At3g13060, Genbank acc. NM_112143, ESTID127j5t7); protein-tyrosine phosphatase (Aunc5, At3g19420, Genbank acc. NM_112829, ESTID122G23T7); translation initiation factor (Aunc6, At3g19760, Genbank acc. NM_112866.3, ESTID137B19T7); helicase (Aunc7, At4g00660, Genbank acc. NM_116291.4, ESTID221A7T7).

### Labelling of array samples

Reverse transcription (RT) of mRNA in the total RNA samples was performed using an oligodT23V primer and with the incorporation of amino-allyl deoxyuridine (aadU, Sigma-Aldrich, Milwaulke, WI). Each RT reaction contained 50 μg total RNA and 10 units Transcriptor Reverse Transcriptase (Roche, Indianapolis, IN) together with 10 μM oligodT23V, 1× first strand buffer (Roche, Indianapolis, IN), 6.6 μM DTT, and nucleotides at 0.5 mM for dA, dG, dC and 0.25 mM for dT and aadU in a total volume of 30 μL. The RNA and oligodT23V were incubated at 70°C for 10 min and cooled to 4°C for 5min. First strand buffer, DTT, nucleotides and enzyme were added and the reaction was incubated for 30 min at 42°C. 1 μL of 20 mM EDTA was added to stop the reaction, and RNA degraded by addition of 1 μL 500 mM NaOH and the sample heated to 70°C for 10 min then neutralised with 1 μL 500 mM HCl.

Apple genomic DNA was isolated from Royal Gala leaves using a Nucleon extraction and purification kit (GE Healthcare). Leaves (1 g) were processed according to the manufacturers instructions and the optional step of adding β-marcaptoethanol was included to limit oxidation of phenolic compounds. DNA was resuspended in 500 μL TE. DNA was sheared by passing through a 26.5 gauge needle 20 times.

Genomic DNA first strand labelling used components of the Radprime DNA Labelling Kit (Invitrogen, Carlsbad, CA) containing Klenow DNA I polymerase and random octamer primers. Each labelling reaction contained 2.5 μg sheared apple genomic DNA and 40 U Klenow together with 1× Radprime buffer (containing primers) and nucleotides at 0.12 mM dA, dG, dC and 0.06 mM dT, aadU in a total volume of 50 μL. Radprime buffer and apple genomic DNA were heated to 95°C for 10 min and cooled to 4°C for 5 min. The nucleotides and enzyme were added and the reaction was incubated for 1 hr at 37°C.

The cDNA and first strand gDNA was ethanol precipitated and resuspended in 5 μL 100 mM Na_2_CO_3 _(pH 9.0). 5 μL Cy3 or Cy5 (Amersham Biosciences, Buckinghamshire, England) in DMSO was added and the sample incubated at room temperature for 2 hr in the dark. Unreacted dye was quenched by addition of 10 μL 4 M hydroxylamine (Sigma, St Louis, MO) and incubation for 10 min at room temperature in the dark. Labelled DNA was purified on PCR Clean-up Columns (Qiagen, Hilden, Germany) and paired samples were pooled. Sample absorbance at 260 nm, 550 nm and 650 nm was measured to determine the amount of labelled DNA and efficiency of Cy3 and Cy5 labelling, respectively. The PCR Clean-up Column purification was repeated once more after pooling to reduce background fluorescence.

### Hybridisation

Labelled samples were hybridised to the microarray slides in an Amersham Lucidea Automated Slide Processor. Slides were pre-washed with 2 × SSC, 0.3% SDS. Samples were injected into the slide chamber together in 3 × SSC, 0.2% SDS, 6% liquid blocking reagent (RPN3601, GE Healthcare, Chalfont St Giles, United Kingdom). Chambers were heated to 45°C and mixed overnight. After hybridisation, arrays were washed with 2 × SSC, 0.3% SDS for 1.2 min and cooled to 30°C, then washed again with 2 × SSC, 0.3% SDS for 1.2 min, 2 × SSC, 0.3% SDS for 2.4 minutes, 0.5 × SSC, 0.2% SDS for 2.4 min (twice) and then once with 0.5 × SSC. Slides were dried by centrifugation and scanned on a GenePix 4000 B Scanner (Axon Instruments). Raw data from scanning of the array slides were captured using GenePix4 (Axon Instruments) and automated spot alignment was augmented with manual checking of each slide to remove substandard spots.

### Normalisation and analysis

All analysis was conducted as described in Schaffer et al. [22], except a one way ANOVA model (y = time) was used. The number of significant differentially expressed genes was examined using a 0.01 threshold using a non-adaptive False Discovery Rate (FDR) control [26]. Expression for each gene was calculated as the mean and standard error, of two technical replicates (dye swap) for both biological replicates for all timepoints (except for 0 DAA where no Rep2 sample was taken).

### Quantitative RT-PCR

Primers for qRT-PCR were designed where possible to overlap the site of the oligo used on the array and qRT-PCR carried out on cDNA made from RNA from the same tissue samples as used for the array experiments. The total RNA extracted for array experiment was used for the qRT-PCR. RNA was treated with DNAase (using the Turbo DNAse kit, Ambion, Austin, TX).

Forward and reverse primers were designed for each qRT-PCR candidate and three control genes. Where possible all primers were designed to span the array oligo, have an optimum temperature of 59°C, GC content 40–60%, amplicon length 100 bp, primer length 20 bp. Primer sequences are shown in Table 9.

**Table 9:** Primers used for qRT-PCR

Genbank Acc.	Forward Primer	Reverse Primer
EE663834	5'-CCATGCAAGTCTTGTTCCTG-3'	3'-TCTTGGAGATGTGGTGAGGA-5'
EB115521	5'-AGGCAGCCTTCTGTCATTG-3'	3'-TCGAATTTCGCATTCTTCTG-5'
EB142488	5'-ACCGGAGCATGGAGACTTT-3'	3'-GGACTAGCCAACATCACACTTG-5'
CN931474	5'-AACTGAGTTGCTTGCAGTCC-3'	3'-TGAGCCGGTTAGTAAAGCAA-5'
CN883166	5'-CCGTTGCGAAGGAAACTACT-3'	3'-CTCCAACAGCAACACCAGAT-5'
CN876582	5'-CGGAGGAAATTCAAGTCTACG-3'	3'-GTTCCGGAATCCATCTTCAT-5'
CN869994	5'-GTTGCTGATCACTCCACCAC-3'	3'-CTTAGTCCTCAATCGGTCAACA-5'
CN878539	5'-GTGAGCACTGTTGAGCCATT-3'	3'-AATGATTCCTTGAGCGGCTA-5'
EB138209	5'-GGAACCCTCAAACCATCATC-3'	3'-GAGTATATCCACATGCCTTGGTC-5'
CN899848	5'-ATCTTCGAGGGAGTGTACGG-3'	3'-TCAACCGGCAAATCCTTAAT-5'
CN894184	5'-TCGAGTCAATTCAGGAAGGAG-3'	3'-GCATATCATGGGCCAAATC-5'
EB140203	5'-CCCACAGTATAATGAGGAAGGA-3'	5'-CCGGTGACTCACATGGAA-3'
CN882408	5'-CCTCCTGATCTGTGGGAATTA-3'	3'-TCAGAGACACTTGGGCTTGT-5'
CN874609	5'-TTCAGCAACGAGGTGTCATT-3'	3'-GAACTTGGTGGAGATGTTGC-5'
CN931994	5'-TGAGGAAGCCATTGTTCAAG-3'	3'-CCTTTGAACATAGAGACCACCA-5'
CN876312	5'-AGTTACGGAGTGTGTTGAGCA-3'	3'-CCAGGTAGTCACGGATGATG-5'
EB140237	5'-AGCTTGACTCTCCACCTCGT-3'	3'-ATGGTGTTTCCATCAGCTTG-5'
CN941270	5'-AAAGCAGAAGCCAGCAATC-3'	3'-CCTTGTGGCTTCGAGTAACC-5'
EB134348	5'-CACTCAGCCAAATCAAGTCG-3'	3'-ACACCCTATGGTCCTCGTTC-5'
EB122025	5'-TGAGGTCGTATGGGAGAAAGA-3'	3'-GCAGTGGTTAGACGGAAGCTA-5'
CN929977	5'-TCAGAATCTCTTGCTAGCTCCTC-3'	3'-CTTGCTCTGGCTACACGAAC-5'
EG631180	5'-GCTGAAGGAGCTGTTGAGAA-3'	3'-TCCCACCACTTGAACAAGAA-5'
CN903005	5'-CGTTGGAGGTTGTGATGATG-3'	3'-CCAACCAACCATCTAACTCTGA-5'
CN946592	5'-AGCCTGAGATACACGGTGGT-3'	3'-TGGTTCCCTCTCCTTTCAAT-5'
CN940056	5'-AACCCTCCTCCTACCTTCCT-3'	3'-AGCACCTATGCGACTGTGAC-5'
CN942749	5'-GTATCATGGTTGGCAATTCG-3'	3'-CTGGAGTCCTTCACCTCGTAT-5'
EB143812	5'-CATTTGCCAGATGGTAGAGC-3'	3'-GATTGCTCACACTCCCAAGA-5'
EG631279	5'-CCGCCGTTTCTTCTATGTATT-3'	3'-CAGAAGCTCCACATCCTTCTT-5'
EB116421	5'-ACCATGTGTCCCTCCTGTG-3'	3'-TCGATCCGATTAAGAATGGAC-5'
EG631302	5'-TTACAGGTGTGCTGCATCAAT-3'	3'-ATTCCAACCGTTGATCACATC-5'
CN893819	5'-CGAAGGTGACACTCCTCTCC-3'	3'-CCGTTAGGTTGCTTGGTAGG-5'
CN911241	5'-CGGAACGAATGATTGATGAG-3'	3'-CATCTGGATTGAGTAGGAACTACC-5'
CN945543	5'-GAAAGTGAGTAATGGTGCTGCT-3'	3'-GACTTGCTTCGGTTAAACACC-5'
CN903467	5'-ATGAGGACGATGAGGATGGT-3'	3'-TCAAGCGTTGTCTCAACTCA-5'
EB124137	5'-AAGCTCAAGCCCTCATGC-3'	3'-GTGGATAAGCACCATTGCAG-5'
EG631379	5'-ATACGAGGGCCCTATGGTT-3'	3'-GAACCTGCAAACTTCAGCAA-5'
CN897963	5'-TACGCCCTCAAGTACAGCAA-3'	3'-CAATTCCTCCGCCTCTTTAT-5'
EE663644	5'-TGGGCTTCGGTACAAGTATG-3'	3'-CACAATCTCCCAGGGATTTC-5'
EE663791	5'-TTGTTCTGCAGCCATTCG-3'	3'-ACGTGGAGAAGGATGAGGAT-5'
EB121923	5'-TGGTGGTAGGGTTGAAACTG-3'	3'-CCCATACCTTCTCAAGGAACA-5'
EE663720	5'-GTTTCATTGGGAGGCTTGA-3'	3'-GCCAGTCCCGAGGACTATAA-5'
EB112628	5'-CCAAGTCGTCGTTGTTGCTA-3'	3'-GGAGCGATGGAGATCTGTCT-5'
EG631202	5'-GAGGCTGCCGTTTCTCTTAT-3'	3'-CGTGCGATTTACCACTCATC-5'
EB114557	5'-GTGCACGTTTCAACACCTTT-3'	3'-GACTGCGGTAGAAGCAACAA-5'
CN884033	5'-CTTGCGAGAGTGTAGCGTTC-3'	3'-AGTAGTCTGCACCCATCATCA-5'
EB144194	5'-CGCCTGCAAGGATTAGATTT-3'	3'-TGTGCTCGGTTCCAGATATT-5'
EE663790	5'-GGTCATGGATTGGAAGGGTA-3'	3'-TGTGACAAACTGCTTACTGCTG-5'
EB156512	5'-CATCCTTCTGGAGTTGAGCA-3'	3'-ATACACCATCCACCCAAACC-5'
EB123469	5'-AGGAACTCCGGAGACTCTTG-3'	3'-AAGCCAACACAGGGATAACA-5'
EB108842	5'-AACTGGCTTGCGTGAGTATG-3'	3'-TCACACCACTCATTGCTTCA-5'

Three independent reverse transcription reactions were performed for each RNA sample. All reactions contained 2 μg of RNA, 2.5 μM oligo(dT)23 V primer and 0.5 mM dNTP mix in 36.5 μL H_2_O. Sample were incubated 5 min at 65°C, 1 min on ice. 1× first strand buffer, 5 mM DTT and 200 Units of Superscript III RT (Invitrogen, Carlsbad, CA) was added, samples incubated 60 min at 50°C and 15 min at 70°C. Replicate reactions were pooled and diluted to 15 ng/uL.

qRT-PCRs were carried out on both biological replicate samples and each reaction was carried out in quadruplicate. Each 20 μL reaction contained 75 ng cDNA, 200 nM forward and reverse primer and 2× SYBR green master mix (Invitrogen, Carlsbad, CA) Amplification was performed using an ABI PRISM 7900 HT sequence detection system (Applied Biosystems, Foster City, CA). Reactions underwent a denaturation stage for 2 min at 94°C, amplified for 40 cycles (15 sec 94°C, 30 sec 59°C, 20 sec 72°C) and a dissociation stage(15 sec 95°C, 30 sec 60°C, 15 sec 95°C). Expression quantification and data analysis were performed in accordance with Snowden et al. [52].

## Authors' contributions

BJJ designed the experiments, designed the oligos used in the array collected samples developed labelling and hybridisation methods, analysed the data and drafted the manuscript. KT carried out labelling and hybridisations, analysis of starch metabolic genes, qRT-PCR of starch genes and assisted with drafting the manuscript. RS assisted in developing hybridisation methods, processed raw array data and developed algorithms for normalisation. RA provided access to tomato array data and carried out analysis of apple data and assisted with the manuscript. LB carried out labelling and hybridisations. RB carried out labelling and hybridisations, qRT-PCR validation and assisted with the manuscript. JHB prepared RNA. RNC provided bioinformatic support and the scripts used to select unigenes and design the oligos used in the array. APG provided sequencing and bioinformatic support to confirm sequences for genes used in the array. SL prepared RNA and assisted with the manuscript. SMcA assisted in design of the experiments and managed the sample collection. FBP assisted in the design of algorithms for normalisation of the data. KCS assisted in design of the experiments and the oligos, analysed data and assisted with the manuscript. SW collected samples. All authors have read the final manuscript without any objections.

## Supplementary Material

Additional file 1 **Array data**. An Excel spreadsheet containing expression data for each array feature. Data is shown as mean, number of data points (n) and standard error (SE) of expression levels for the two biological and two technical replicates for all samples except 0 DAA where only one biological replicate was sampled. In some cases one or more data points were excluded from analysis for technical reasons in which case the mean and standard error is calculated from the remaining data. Raw data is lodged with GEO. Click here for file
